# Detection of increased serum miR-122-5p and miR-455-3p levels before the clinical diagnosis of liver cancer in people with type 2 diabetes

**DOI:** 10.1038/s41598-021-03222-x

**Published:** 2021-12-09

**Authors:** Heung Man Lee, Willy Kwun Kiu Wong, Baoqi Fan, Eric Siu Lau, Yong Hou, Chun Kwan O, Andrea On Yan Luk, Elaine Yee Kwan Chow, Ronald Ching Wan Ma, Juliana Chung Ngor Chan, Alice Pik Shan Kong

**Affiliations:** 1 Department of Medicine and Therapeutics, The Chinese University of Hong Kong, Prince of Wales Hospital, Shatin, N.T., Hong Kong SAR China; 2 Li Ka Shing Institute of Health Sciences, The Chinese University of Hong Kong, Prince of Wales Hospital, Shatin, Hong Kong SAR China; 3 Hong Kong Institute of Diabetes and Obesity, The Chinese University of Hong Kong, Prince of Wales Hospital, Shatin, Hong Kong SAR China

**Keywords:** Cancer, Predictive markers

## Abstract

People with type 2 diabetes (T2D) have increased cancer risk. Liver cancer (LC) has a high prevalence in East Asia and is one of the leading causes of cancer death globally. Diagnosis of LC at early stage carries good prognosis. We used stored serum from patients of Hong Kong Diabetes Register before cancer diagnosis to extract RNA to screen for microRNA markers for early detection of LC in T2D. After screening with Affymetrix GeneChip microarray with serum RNA from 19 incident T2D LC (T2D-LC), 20 T2D cancer free (T2D-CF) and 20 non-T2D non-cancer patients, top signals were validated in a 3-group comparison including 1888 T2D-CF, 127 T2D-LC, and 487 T2D patients with non-liver cancer patients using qPCR. We detected 2.55-fold increase in miR-122-5p and 9.21-fold increase in miR-455-3p in the T2D-LC group. Using ROC analysis, miR-122-5p and miR-455-3p jointly predicted LC with an area under the curve of 0.770. After adjustment for confounders, each unit increase of miR-455-3p increased the odds ratio for liver cancer by 1.022. Increased serum levels of miR-122-5p and miR-455-3p were independently associated with increased risk of incident LC in T2D and may serve as potential biomarkers for early detection of LC in T2D.

## Introduction

Diabetes and cancer are complex diseases sharing many common biological pathways. Individuals with type 2 diabetes (T2D) have 1.3 to threefold increased risk for most cancers^1–3^. Due to aging and improved survival from cardiovascular events, cancer is the leading cause of death and accounts for one in four deaths in Hong Kong Chinese with T2D^4^. Epidemiological studies support close associations between hyperglycemia and risk for all-site cancer in T2D^5,6^. In 1995, we established a prospective cohort, the Hong Kong Diabetes Register (HKDR)^7^ as an ongoing quality improvement program to evaluate causes and consequences of diabetes in Chinese people. Using total cholesterol (TC) level, white blood cell (WBC) count, age and smoking status, we have developed and validated an all-cancer risk score for T2D with an area under the receiver operating characteristic (ROC) curve of up to 0.71^8^. We also reported that every 1% increase in glycated hemoglobin (HbA_1c_) was associated with 18% increased hazard ratio in all-cancer risk^5^. In the HKDR, half of the cancer events in T2D occurred in the gastrointestinal system including liver^9^. In patients with T2D and chronic hepatitis B virus (HBV) infection, the hazard ratio of hepatocellular carcinoma, the major form of liver cancer (LC) was 75.0 in those with HbA_1c_ ≥ 7% versus 3.7 in those with HbA_1c_ < 7% using non-HBV carriers with T2D and HbA_1c_ < 7% as control^10^.

Due to the silent nature of LC, clinical diagnosis is often delayed resulting in poor prognosis^11^. Despite intensive research, there has been limited progress in developing efficacious methods to detect and diagnose LC early^12^. Recent studies indicated that serum microRNA (miRNA) might be used as an early marker for some cancers^13^. Here, miRNA is a family of small noncoding RNA with 19–28 nucleotides that can regulate gene expression. Generally, miRNA binds to the target sequence at the 3’ untranslated region of mRNA to suppress gene expression through post-transcriptional mechanisms^14^. One miRNA can have multiple targets in different regulatory pathways, such as cell proliferation, gene expression, apoptosis and cancer development^15^.

Altered expression of miRNA has been reported in many diseases^15^ including cancer^16,17^. Given its upstream regulatory role in tumorigenesis, there are ongoing efforts to develop a miRNA signature for cancer typing^18^ while the circulating miRNA might be used for cancer detection^17^. Quantitative real-time PCR (qPCR) has been used to quantify serum miRNA which exhibited specific expression patterns with lung and colorectal cancers^13,19^. As serum miRNA levels are stable and reproducible, qPCR measurement of serum miRNA level is a potential non-invasive method for early cancer detection^13^.

Based on HKDR cohort^7^, we explored the clinical utility of miRNA for predicting LC by measuring their levels in stored serum samples before the clinical diagnosis of LC in T2D patients enrolled in the HKDR.

## Methods

All procedures performed in this study were carried out in accordance with the guidelines and regulations of The Chinese University of Hong Kong including use of human samples and disposal of biological and chemical wastes.

### Samples

The HKDR was established in 1995 as an ongoing research-driven quality improvement program with a weekly enrolment of 30 to 50 ambulatory patients with diabetes referred from community and hospital-based clinics who underwent structured assessment using standard protocols^7^. The samples of the study were selected from HKDR. Informed consent have been obtained from all participants of the study. The use of serum samples from HKDR for this study was approved by the Joint Chinese University of Hong Kong–New Territories East Cluster Clinical Research Ethics Committee (CREC Ref. No.: 2016.213).

Hong Kong has 7 million population, mainly Southern Chinese. It has a universal healthcare system governed by the Hong Kong Hospital Authority, established since 1990, which operates a territory-wide network of hospitals and clinics that share a single electronic medical record (EMR) system using a unique identifier. All hospitalization records were recorded using the International Classification Code (ICD-9). The HKDR was set up at the Prince of Wales Hospital with a catchment population of 1 million. The serum samples used in this study were selected from the HKDR based on their clinical profiles at enrolment. The clinical outcomes of all patients were censored on June 30th 2017 and LC were identified by hospitalization records based on ICD-9 code 155.

**Figure 1:**
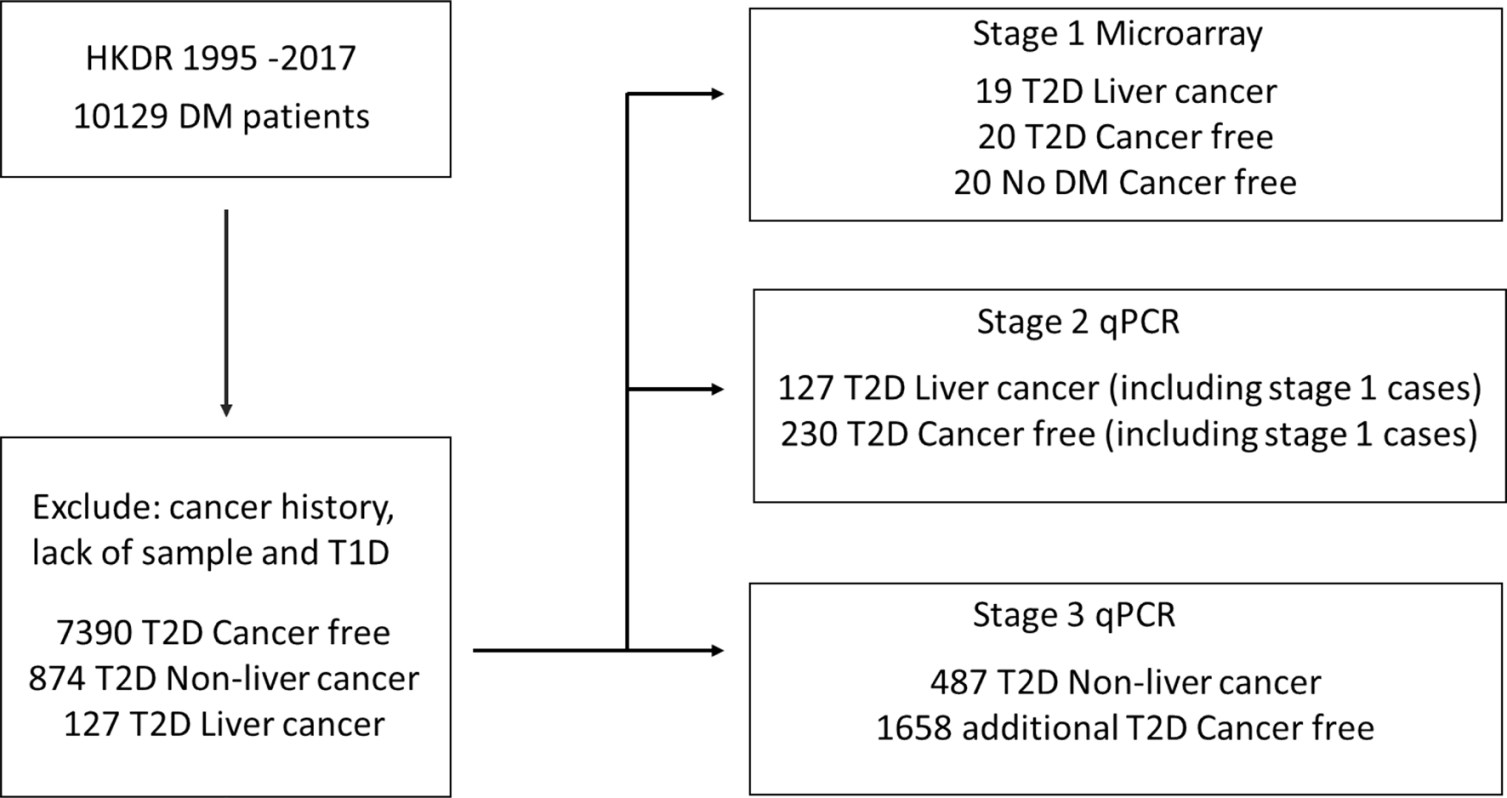
Study flow.

Between 1995 and 2017, 10,129 patients were enrolled to the HKDR accompanied by a biobank. After excluding patients with type 1 diabetes (defined as ketotic presentation or continuous requirement of insulin within one year of diagnosis) and those with prior history of any cancer and lack of stored samples, 8391 patients were included in our sample selection which included 127 T2D patients diagnosed with LC after registration (Fig. 1). The medical records of each patient with LC were reviewed to confirm the primary diagnosis of LC, ascertain their carrier status for hepatitis B surface antigen (HBsAg) and exclude cases due to metastatic cancer.

The study consisted of 3 stages. In the stage 1 microarray study, we selected 19 T2D patients with serum samples collected 0.5 to 6 years before their first diagnosis with LC (T2D-LC) and 20 T2D patients who were cancer-free (T2D-CF) matched for age, sex, disease duration and body mass index (BMI) as controls (Table 1). We also included 20 healthy subjects without incident diabetes and cancer from a prospective community health promotion project^20^ (non-T2D-CF). In stage 2 qPCR study, 127 T2D-LC and 230 T2D-CF patients (including those in stage 1) matched for age, sex, disease duration and BMI (Supplementary Table S1) were analysed to select the top miRNAs associated with LC. In stage 3 study, we selected an additional 1658 T2D-CF patients and 487 patients with other non-liver cancer (T2D-NLC) (Supplementary Table S2). We combined patients in stage 2 and stage 3 to evaluate the performance of the selected miRNA markers in T2D-LC (n = 127) and T2D-NLC group (n = 487) compared with T2D-CF patients (n = 1888).

**Table 1:** Characteristics of the patients selected for stages 1, 2 and 3 of the study.

	Stage 1 microarray study cases				Stage 2 and stage 3 qPCR Validation cases			
	T2D without cancer	T2D with liver cancer	Non T2D without cancer	*P* value	T2D without cancer	T2D with liver cancer	T2D with other cancer	*P* value
Cases (N)	20	19	20		1888	127	487	
Sex (F:M)	10 : 10	9 : 10	10 : 10		909 : 979	27 : 100	254 : 233	
Age (years)	61.40 ± 9.62	62.42 ± 9.55	50.80 ± 3.53	< 0.001	57.69 ± 12.27	58.59 ± 10.33	63.12 ± 10.12	< 0.001
Disease duration of diabetes (years)	6.80 ± 5.06	6.58 ± 5.06	NA	0.887*	7.12 ± 6.69	6.35 ± 5.36	7.17 ± 6.64	0.388
Body mass index (BMI)	23.39 ± 3.71	23.43 ± 3.83	23.21 ± 3.51	0.981	25.30 ± 4.55	24.21 ± 3.22	25.42 ± 4.22	0.020
HbA_1c_ level (%)	8.32 ± 1.79	7.56 ± 1.89	NA	0.207*	7.64 ± 1.75	7.79 ± 1.79	7.61 ± 1.74	0.584
Fasting plasma glucose (mmol/L)	9.43 ± 0.83	8.20 ± 3.23	4.95 ± 0.42	< 0.001	8.63 ± 3.28	8.42 ± 3.10	8.67 ± 3.29	0.742
Total cholesterol (mmol/L)	5.06 ± 1.12	4.93 ± 1.18	5.21 ± 0.85	0.713	5.24 ± 1.15	4.89 ± 1.22	5.19 ± 1.04	0.004
WBC Count (10^9^ cells/L)	7.39 ± 1.69	6.36 ± 1.47	5.93 ± 1.09	0.008	7.40 ± 3.69	6.65 ± 2.51	7.23 ± 2.04	0.058
Years before cancer diagnosis		2.28 ± 1.54				6.10 ± 4.89	7.19 ± 5.15	0.068^#^
Follow-up period (years)	16.39 ± 3.96	14.39 ± 4.19		0.134*	16.12 ± 3.41	16.12 ± 3.50	16.52 ± 3.36	0.071
All-site cancer risk score	− 1.26 ± 0.73	− 1.03 ± 0.86		0.375*	− 1.25 ± 0.96	− 0.96 ± 1.06	− 1.00 ± 0.92	< 0.001
Tested for HBsAg (%)	3 (15.0%)	19 (100.0%)			653 (34.6%)	119 (93.7%)	198 (40.7%)	
Tested positive for HBsAg (%)	0 (0%)	12 (63.2%)			80 (4.2%)	77 (60.6%)	15 (3.1%)	
Ex- or current alcohol drinker (%)	6 (30.0%)	6 (31.6%)			428 (22.7%)	52 (40.9%)	110 (22.6%)	

Data were presented as mean ± standard deviation. For stage 1 microarray study, the differences among group means were compared by analysis of variance, ANOVA excepted stated otherwise. *T2D without cancer were compared to T2D with liver cancer using the t-test. ^#^T2D with liver cancer were compared to T2D with other cancers using the t-test.

*BMI* body mass index, *F* female, *HbA*_1c_ glycated haemoglobin, *HBsAg* hepatitis B surface antigen, *M* male, *N* number, *NA* not applicable or not available, *T2D* type 2 diabetes, *WBC* white blood cell.

### Serum RNA extraction

RNA were extracted from serum using the Trizol reagent with modified procedures. In brief, 0.15 ml of serum was extracted with 0.75 ml of Trizol reagent. Synthetic RNA oligonucleotides with identical sequence to ath-miR-172a and cel-miR-39-3p respectively were spiked in during the extraction. Glycogen was added to facilitate RNA precipitation. After alcohol precipitation and washing, the RNA pellets were suspended in 15 µl of RNase free water and stored at -80 °C before use.

### Affymetrix microarray assay

The Affymetrix Gene Chip miRNA 4.0 microarray was used to discover serum miRNA markers for LC. We used 240 ng of serum-extracted RNA from each stored sample as template for labelling. All labelling, hybridization and washing procedures were carried out by the staff of the Core Laboratory of the Li Ka Shing Institute of Health Sciences, The Chinese University of Hong Kong following standard protocols. The microarray data were analysed using the software Transcriptome Analysis Console v.4.0.2 from Affymetrix (Santa Clara, CA).

### Quantitative real-time PCR (qPCR)

The Taqman® Advanced miRNA Assays (ThermoFisher Scientific) were used for qPCR assays to measure serum miRNA levels. For the reverse transcription step, 2 µl of serum miRNA was converted to cDNA using the Taqman® Advanced miRNA cDNA Synthesis Kit (ThermoFisher Scientific) with standard procedures. The first strand cDNA was pre-amplified for 16 cycles and diluted 1:8 for the qPCR assays using the ABI QuantStudio 12 K Flex OpenArray® real-time PCR instrument. As an internal control, each reverse transcription reaction contained equal amount of cel-miR-54-3p for adjustment of variations in the assay. We included a control RNA sample for each batch of reverse transcription in 96-well microplates for normalization of the qPCR assays during data analysis. We used these controls to adjust for batch-to-batch variations during reactions for normalization to a common standard for comparison.

For qPCR assays, it is common to use the expression of a reference gene that shows no difference among the different experimental groups as internal control for normalization^21^. The manufacturer of the Taqman™ Advanced miRNA assays recommended several targets as internal control for normalization. (https://www.thermofisher.com/search/results?query=miRNA-controls-WhitePaper&focusarea=Search%20All). We examined these miRNAs in our microarray study to select suitable internal control (Supplementary Table S3). Among these suggested miRNAs, miR-451a, miR-361-5p and miR-186-5p showed 5% or less difference between the T2D-CF and T2D-LC groups. The remaining miRNA (miR-191-5p, miR-423-5p, miR-320a and miR-26a-5p) were not selected due to large between-group variations. We ran the qPCR assays for miR-451a, miR-186-5p and miR-361-5p in the stage 2 samples. We could detect miR-186-5p in over 98% of samples. The respective detection rates were 81% and 92% for miR-361-5p and miR-451a. Serum level of miR-186-5p was slightly lower in the T2D-LC than the T2D-CF group, albeit not significant and was used as the reference for normalization (Supplementary Fig. S2 and Supplementary Table S5).

### Data analysis and statistical analysis

The microarray data were analysed using the software Transcriptome Analysis Console v.4.0.2 from Affymetrix. The miRNA levels were normalized with quantile normalization. The miRNA levels were compared with the built-in statistical analysis of the software. One-way Analysis of Variance (ANOVA) with false discovery rate (FDR) correction was used to compare the normalized miRNA levels among the three groups (T2D-LC, T2D-CF, non-T2D-CF). We compared the miRNA levels of T2D-LC and T2D-CF groups using F test with FDR correction.

The qPCR results were analysed by IBM Statistical Package for Social Sciences v.25 (Armonk, NY). The Student’s t-test was used for 2-group comparison of miRNA levels. If the Levene’s test showed unequal variance between the 2 groups, the Welch’s t-test was used instead. For 3-group comparison, one-way ANOVA was used. The area under the curve (AUC) of the receiver operating characteristic (ROC) curve analysis, expressed in 95% confidence interval (CI), was used to test the validity, sensitivity and specificity of the serum miRNA levels in predicting LC. The optimal point for the ROC line was determined by Youden’s index^22^. We applied logistic regression analysis to test independent association of LC with serum miRNA levels after adjusting for other risk factors and miRNAs. For the ROC analysis and logistic regression analysis, cases with missing values were excluded.

## Results

### Use of microarray to discover serum miRNA associated with LC in T2D

**Figure 2:**
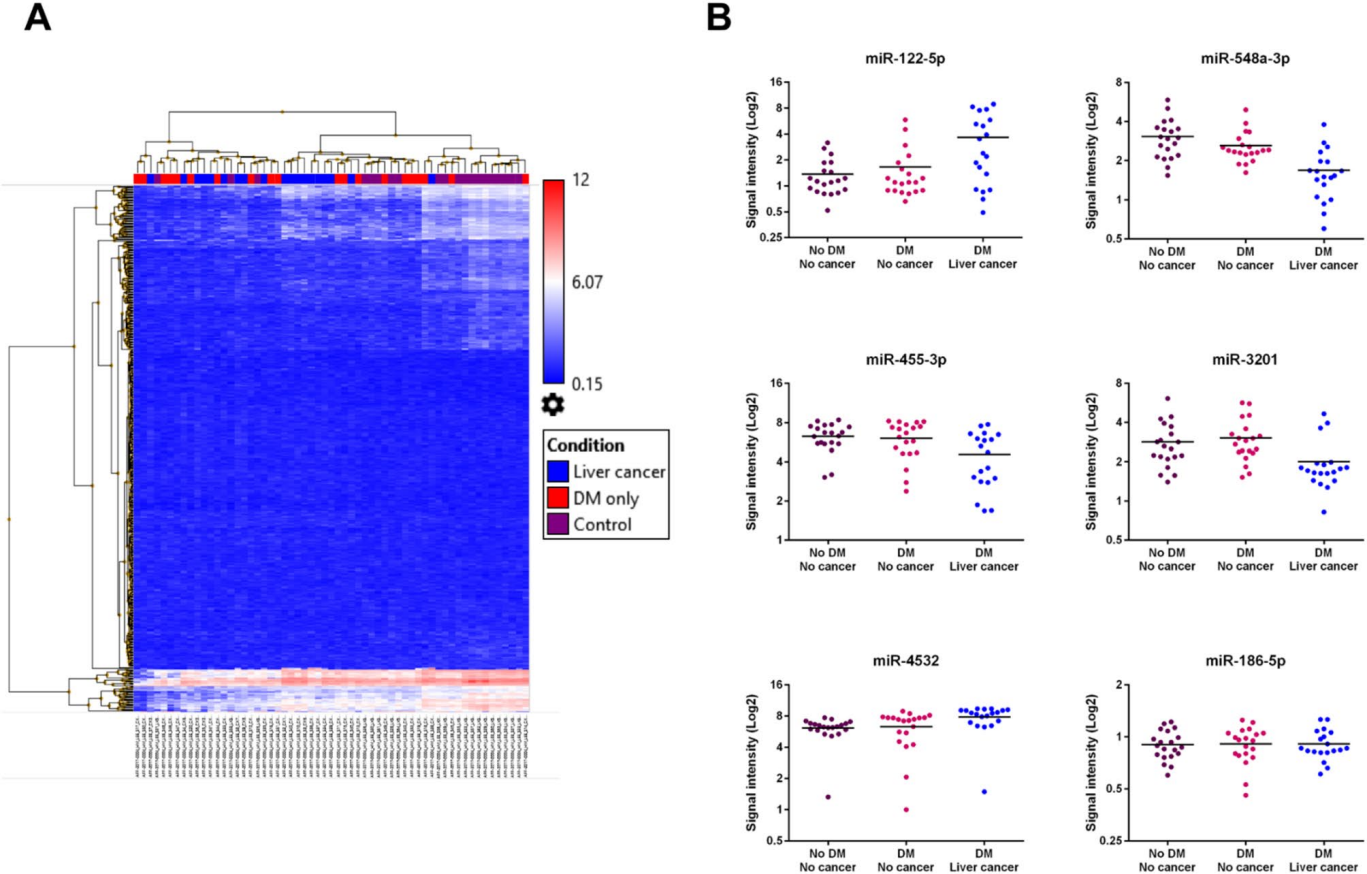
Summary of microarray analysis of serum miRNA. (**A**) Hierarchical analysis of clustering of serum miRNA levels in non-T2D-CF (Control, purple), T2D-CF (DM only, red) and T2D-LC (Liver cancer, blue) samples. (**B**) Individual levels of the miR-122-5p, miR-455-3p, miR-4532, miR-548a-3p, miR-3201 and miR-186-5p from the microarray were shown. The mean of each group was shown as a horizontal line along the individual data points. The signal intensity was shown in log2 scale.

In stage 1, we selected 19 T2D-LC and 20 matched T2D-CF patients as well as 20 non-T2D-CF subjects. The T2D-CF and T2D-LC group were generally well matched with the latter group having shorter disease duration and follow up period as well as lower glycemic indexes, WBC count and all-site cancer risk score (a low score indicating high cancer risk)^8^ (Table 1). Using the Affymetrix Gene Chip miRNA 4.0 microarray, 4603 human miRNAs or miRNA precursors were detected in our samples. Principal component analysis showed no clustering of the cases from the same group (Supplementary Fig. S1). Amongst the detected miRNAs, 519 human miRNA showed significant difference (*P* < 0.05) in the 3-way analysis. Figure 2A showed the hierarchical clustering of these miRNA in the 519 samples. Further comparison from these 519 miRNAs showed that 145 miRNAs were significantly different (*P* < 0.05) between the T2D-LC and T2D-CF groups (Supplementary Table S4) with 5 miRNAs showed at least 50% fold change. Among these 5 miRNAs, miR-548a-3p, miR-3201, and miR-455-3p showed over 50% reduction and miR-122-5p and miR-4532, showed over twofold increase in the T2D-LC group compared with T2D-CF and non-T2D-CF group (Fig. 2B, Table 2). These five miRNAs were selected for qPCR validation in additional serum samples.

**Table 2:** List of microRNA showing significant difference in patients with type 2 diabetes and liver cancer.

	Stage 1 Microarray study						Stages 2 + 3 Validation by qPCR						
microRNA	^†^Non-T2D without cancer	^†^T2D without cancer	^†^T2D with liver cancer	Fold Change (T2D with liver cancer vs T2D without cancer)	*P* value (T2D with liver cancer vs. T2D without cancer)	ANOVA P value	^#^T2D without cancer (N = 1888)	^#^T2D with liver cancer (N = 127)	^#^T2D with other non-liver cancer (N = 487)	Fold Change (T2D with liver cancer vs T2D without cancer)	*P* value (T2D with liver cancer v. T2D without cancer)	*P value* (T2D with liver cancer vs T2D without cancer plus T2D with non-liver cancer)	ANOVA P value
miR-548a-3p	2.91 ± 1.10	2.35 ± 0.77	1.53 ± 0.77	−1.76	0.0020	3.98E-5							
miR-3201	2.55 ± 1.17	2.68 ± 1.19	1.66 ± 0.98	−2.02	0.0050	0.0125							
miR-122-5p	1.12 ± 0.70	1.06 ± 1.35	2.65 ± 2.87	3.01	0.0014	0.0005	32.86 ± 3.29	84.00 ± 23.11	38.46 ± 12.67	2.55	0.030*	0.035*	0.010
Log of miR-122-5p							0.45 ± 0.02	1.19 ± 0.07	0.43 ± 0.05		< 0.001	< 0.001	0.011
miR-455-3p	6.49 ± 1.47	6.27 ± 1.84	4.58 ± 2.03	−3.22	0.0107	0.0075	1.02 ± 0.31	9.39 ± 5.85	0.59 ± 0.26	9.21	0.156*	0.152*	< 0.001
Log of miR-455-3p							−2.55 ± 0.03	−1.42 ± 0.14	−2.31 ± 0.07		< 0.001	< 0.001	< 0.001
miR-4532	6.35 ± 1.32	7.23 ± 2.15	8.40 ± 1.87	2.26	0.0117	0.0085	305.44 ± 100.39	3833.09 ± 2888.09	181.35 ± 72.92	12.55	0.225*	0.221*	< 0.001
Log of miR-4532							0.18 ± 0.03	0.68 ± 0.11	0.48 ± 0.05		< 0.001	< 0.001	< 0.001

^†^The expression levels from the microarray study were presented as mean ± standard deviation in log2 scale.

^#^The qPCR data were presented as mean ± standard deviation.

*Unpaired t-test with Welch’s correction was used for the analysis.

### Validation of LC associated serum miRNA by qPCR in 2-group comparison

From the microarray study, miR-548a-3p, miR-3201, miR-455-3p, miR-122-5p, and miR-4532 (Table 2) were selected for validation using qPCR. We included all 127 T2D-LC patients and expanded the T2D-CF patients from 20 in the stage 1 study to 230 T2D-CF patients for qPCR validation. The 2 groups were well matched for age, sex, BMI, and disease duration, except for a lower WBC count, TC and all-cancer risk score in the T2D-LC group (supplementary Table 1). The serum levels of miR-122-5p and miR-455-3p were higher in the T2D-LC group than T2D-CF group (Supplementary Fig. S2). The serum level of miR-4532 was also higher in the T2D-LC group, albeit not significant. There was no between-group difference for serum levels of miR-3201 and miR-548a-3p (Supplementary Table S4).

Using miR-186-5p as internal control for normalization, the qPCR results were analyzed using the ΔΔCt method. The results were shown in Supplementary Fig. S3. Because the serum miRNA levels were not in normal distribution, we also compared the logarithm of the miRNA levels. The difference of log-miR-122-5p, log-miR-455-3p, log-miR-4532 and log-miR-3201 were statistically significant. The level of miR-122-5p showed a significant 14.06-fold increase in patients with T2D-LC compared with T2D-CF group. The respective fold difference for log-miR-455-3p and log-miR-4532 were 1.9 and 7.2, albeit not significant (Supplementary Table S5).

### Serum miR-122-5p, miR-455-3p and miR-4532 levels in T2D-LC in 3-group comparison

**Figure 3:**
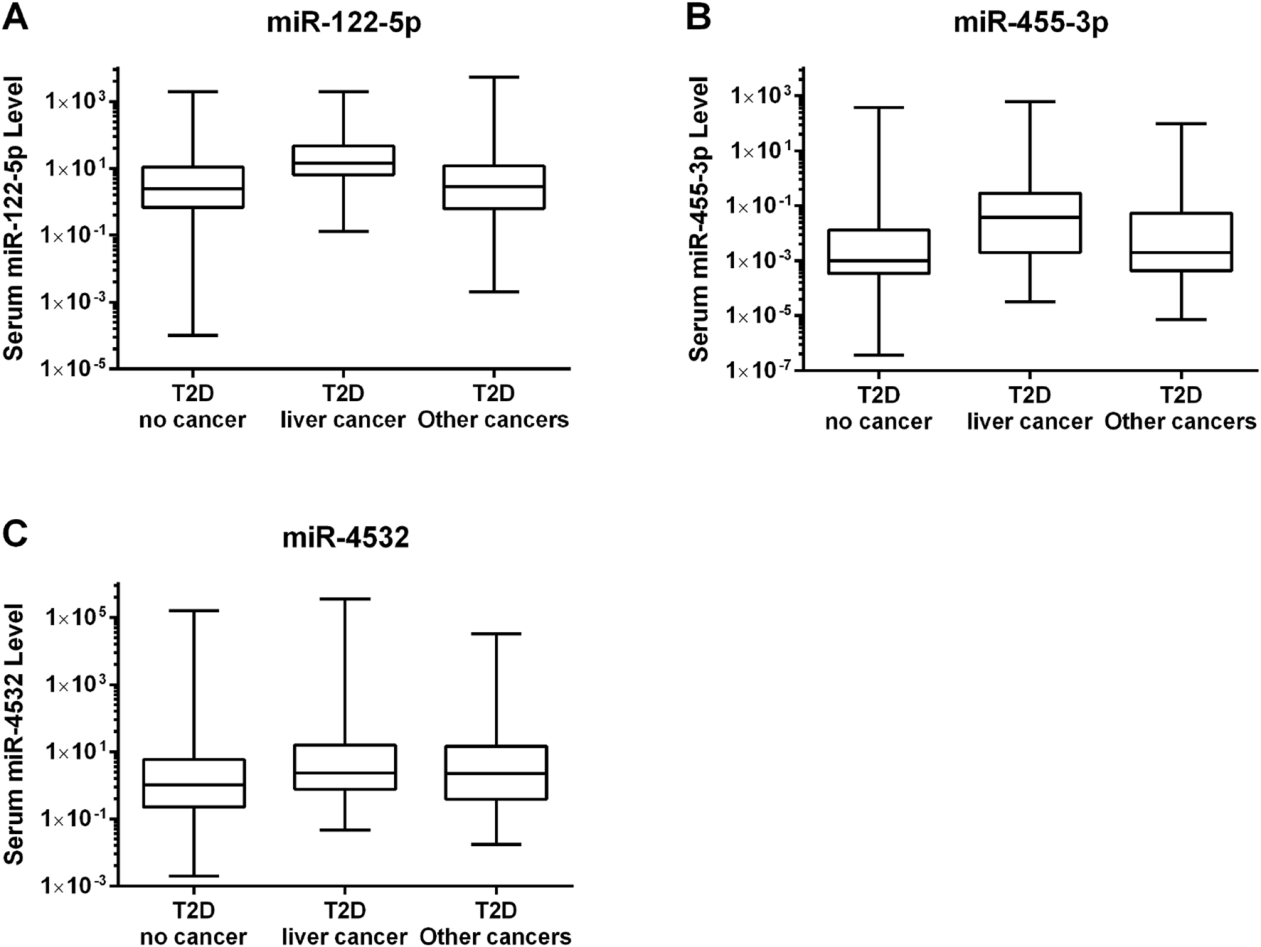
Expression of serum miRNA levels in T2D patients. Serum levels of (**A**) miR-122-5p, (**B**) miR-455-3p and (**C**) miR-4532 are shown in box and whiskers in T2D no cancer (T2D-CF), T2D liver cancer (T2D-LC) and T2D other cancer patients (T2D-NLC). The box represents the 25th to 75th percentile range and the whiskers represent the minimum to maximum. The horizontal line in the box represents the median.

Based on the results of the stage 2 qPCR validation, miR-122-5p, miR-4532, and miR-455-3p were tested in an expanded case–control cohort of 2145 T2D patients. We selected 1658 T2D-CF patients and 487 T2D-NLC patients who had other cancer types. Both groups had similar characteristics except for older age and lower all-cancer risk score in the T2D-NLC group (Supplementary Table S2). We included the T2D patients in stage 1 and stage 2 giving a total of 1888 T2D-CF patients, 127 T2D-LC and 487 T2D-NLC patients in a 3-group comparison. (Table 1). Figure 3 showed the serum levels of miR-122-5p, miR-455-3p and miR-4532 in these three groups. Using ANOVA, the serum levels of miR-455-3p, miR-122-5p, and miR-4532 were different amongst the 3 groups, reaching significant for miR-122-5p between T2D-CF and T2D-LC group. Using miR-186-5p as internal control for normalization, the logarithmic values amongst all three miRNAs were significantly different in line with the serum levels (Table 2).

**Figure 4:**
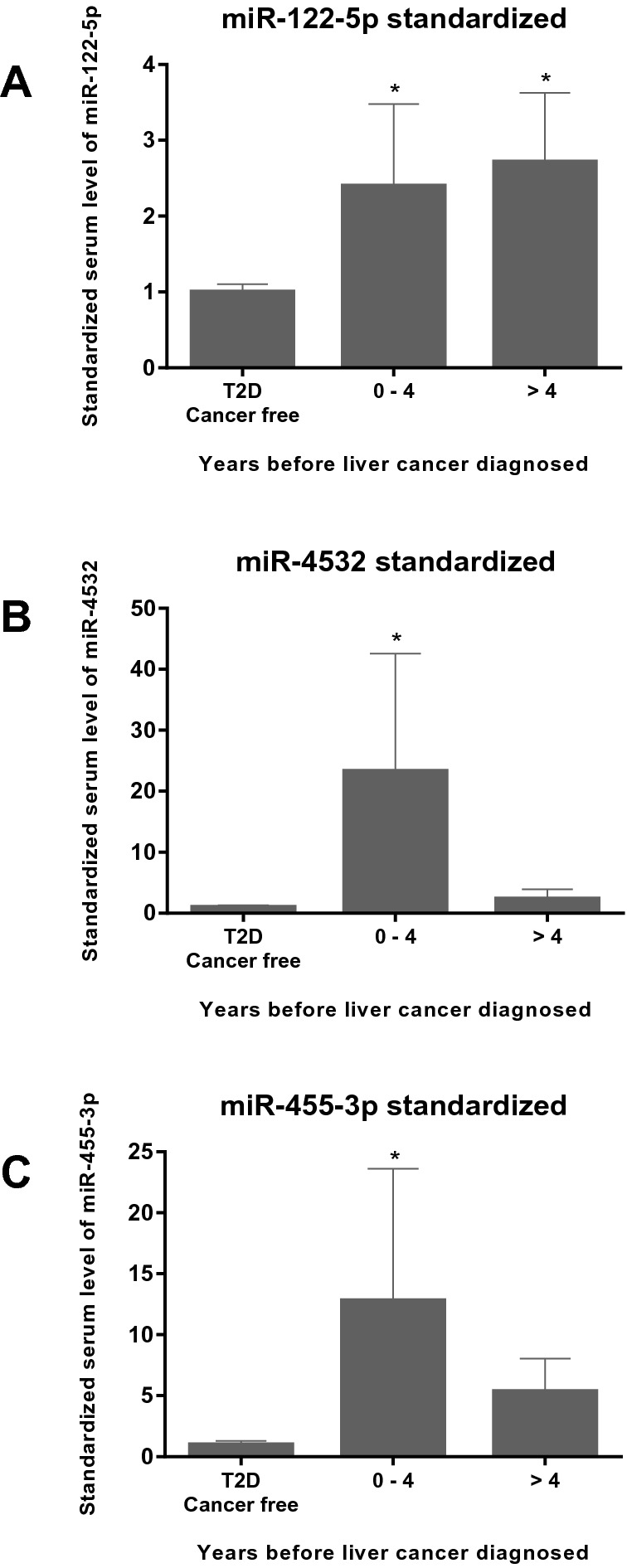
Expression of serum miRNA levels in T2D liver cancer patients. Serum levels of miR-122-5p (**A**), miR-4532 (**B**) and miR-455-3p (**C**) are shown. The T2D liver cancer patients were categorized by the length of the period between the enrolment dates in HKDR and their first liver cancer diagnosis. The serum levels of the miRNAs are standardized to the mean serum levels of the T2D cancer free patients. The bar chart with error bars represent the mean ± standard deviation. *P < 0.05 when comparing to the T2D cancer free group.

The serum samples of the 127 T2D-LC patients analysed in this study were collected 0.2 to 18.8 years before LC was diagnosed. We compared the serum levels of miR-122-5p, miR-4532 and miR-455-3p at enrolment during the lead time before diagnosis of LC versus that in the T2D-CF group. Significant increase in serum miR-122-5p levels was detected zero to four years before LC diagnosis. In patients followed up for more than four years, increased level was also detected before diagnosis. Similarly, significant increase in serum miR-4532 and miR-455-3p levels were detected in a lead time ranging from zero to four years before the diagnosis of LC (Fig. 4).

Chronic hepatitis B viral (HBV) infection and chronic use of alcohol are known risk factors of LC^10,11^. We extracted HBsAg information from the medical records of all patients included in the analysis. Amongst the 127 T2D patients with LC, 119 had HBsAg tested and of these, 77 were positive (65%). In the remaining 2375 T2D patients, 851 had HBsAg tested and of these, 95 were positive (11%). Amongst patients with available HBsAg results, serum levels of miR-122-5p was higher in the HBsAg carriers than the non-carriers. The serum levels of miR-455-3p and miR-4532 were similar between the two groups (Supplementary Table S6). In the whole group, 590 patients were considered regular alcohol users based on consumption in the last 12 months. Serum levels of all 3 miRNAs were similar between current/ex alcohol users and non-users (Supplementary Table S7).

**Figure 5:**
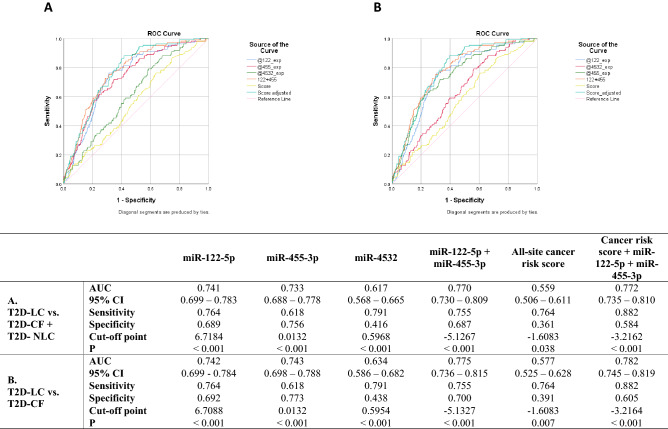
Receiver operating characteristic (ROC) analysis of serum microRNA (miRNA) level in type 2 diabetes (T2D) with liver cancer cases versus T2D without cancer cases and T2D non-liver cancer cases. The miR-186-5p normalized serum levels of miR-122-5p (@122_exp), miR-455-3p (@455_exp) and miR-4532 (@4532_exp) were analysed using SPSS v.25. The sum of Log values serum level of miR-122-5p and miR-455-3p (122 + 455) and the all-site cancer risk score and the sum of 122 + 455 and all-site cancer risk score (Score adjusted) were included for comparison. The serum levels of T2D liver cancer group were tested against the T2D cancer-free group and T2D with other cancer group combined (**A**) and the T2D cancer-free group only (**B**). The diagonal reference line was shown for comparison. The summaries of area under the curve (AUC) with 95% confidence intervals (95% CI), optimal sensitivity and specificity and *P* values were shown below.

We ran ROC analysis with serum miRNA levels and all-site cancer risk score derived from the HKDR based on age, TC, WBC count and smoking status^8^ to predict LC. We combined T2D-CF and T2D-NLC as control group versus T2D-LC group. The AUC for different combinations of all-site cancer risk score and miRNAs ranged from 0.559 to 0.772. The AUC for predicting LC was 0.741 (0.699–0.783, *P* < 0.001) for miR-122-5p alone and 0.733 (0.688–0.778, *P* < 0.001) for miR-455-3p alone. The combined use of miR-122-5p and miR-455-3p increased the AUC to 0.770 (0.730–0.809, *P* < 0.001). This further increased to 0.772 (0.735–0.810, *P* < 0.001) by including the all-site cancer risk score with the specificity falling from 0.687 to 0.584 whilst sensitivity increasing from 0.755 to 0.882 (Fig. 5A). If the ROC analysis was run after excluding the T2D-NLC group, the serum levels of miR-122-5p and miR-455-3p combined yielded an AUC of 0.775 (0.736–0.815, *P* < 0.001) and 0.782 (0.745–0.819, *P* < 0.001) when the risk score was included (Fig. 5B).

We ran logistic regression analysis to examine the independent risk association of LC with serum levels of validated miRNAs. Using T2D-CF patients as control, each unit increase of serum miR-455-3p level increased the odds ratio (OR) of LC by 1.021 (1.000–1.043) (Table 3, model 1, *P* = 0.050) after adjusting for miR-122-5p, HBsAg, alcohol use and all-site cancer risk score^8^. Using both T2D-CF and T2D-NLC group as control, the OR was 1.022 (1.000–1.042, *P* = 0.026; Table 3, model 2).

**Table 3:** Association of serum miR-455-3p with T2D liver cancer using logistic regression analysis.

	Beta	*P* value	Odds ratio	95% C.I	
				Lower	Upper
**Model 1** T2D patients with liver cancer versus T2D patients without cancer Adjusted for miR-122-5p, all-site cancer risk score, HBsAg status and use of alcohol	0.021	0.050	1.021	1.000	1.043
**Model 2** T2D patient with liver cancer versus T2D patients without liver cancer (T2D-CF plus T2D-NLC) Adjusted for miR-122-5p, cancer risk score, HBsAg status and use of alcohol	0.022	0.026	1.022	1.003	1.042

## Discussion

In this 3-stage study using a prospective cohort of T2D patients, we selected patients who developed LC with a lead time of 0.2–18.8 years (6.1 ± 4.9, mean ± SD years) between enrolment and clinical diagnosis as cases. Control subjects included patients with T2D who remained cancer-free or had other cancer types during a mean observation period of up to 16 years. In a small discovery cohort, we applied the Affymetrix GeneChip microarray and discovered five miRNAs associated with LC. Of these, miR-122-5p, miR-455-3p, and to a lesser extent miR-4532 were identified as potential markers using qPCR. In the third stage involving more than 2000 T2D patients, we confirmed that increased serum levels of these three miRNAs were detectable in stored serum zero to four years before the clinical diagnosis. The ROC analysis indicated that miR-122-5p and miR-455-3p had the best performance with respective AUC of 0.741 and 0.733. Other researchers had reported an AUC of 0.77 for miR-122 with prevalent liver cancer^23^. The AUC of these miRNA levels were comparable or higher than that of 0.71 for the all-site cancer risk score based on clinical and biochemical parameters in the HKDR^8^. Due to the smaller sample size, the AUC for this clinical risk score declined to 0.559 in this study. On logistic regression analysis, miR-455-3p remained independently associated with increased risk of LC after adjusting for all-site cancer risk score, miR-122-5p, HBsAg and alcohol use. This lead time had made miR-455-3p and miR-122-5p potential biomarkers for regular surveillance to detect LC in T2D patients.

The poor prognosis of LC is in part due to delayed diagnosis^11^. To date, there is limited success in developing tests to identify high risk subjects for LC for undergoing definitive tests such as imaging^12^. Several studies suggested that serum miRNA might be a marker for cancers^13^. Among the serum miRNA identified from prevalent liver cancers^17^, only miR-122-5p showed increased serum level in our study (Supplementary Table S8). Liver has high levels of expression of miR-122-5p which is a key regulator in cholesterol and fatty acid metabolism^24,25^. Silencing miR-122-5p in mice resulted in steatohepatitis, liver fibrosis and high incidence of hepatocellular carcinoma. These pathological changes were attenuated with restoration of the expression of miR-122-5p^26^. In our study, increased serum miR-122-5p level was detected zero to four years before their first diagnosis with LC. Experimental studies supported an inhibitory effect of miR-122-5p on LC cells^24,27,28^. Thus, its reduced expression in LC with high circulating level raised several possibilities. These included disposal of miR-122-5p from LC cells to the extracellular space, secretion of miR-122-5p by normal hepatocytes as a defence mechanism or its release due to necrotic or apoptotic cell death although further experimental studies are needed to test these hypotheses.

Similarly, we detected increased serum levels of miR-4532 and miR-455-3p zero to four years before the diagnosis of LC. While miR-455-3p had been reported to be an early marker for Alzheimer’s disease^29^ and breast cancer^30^, this miRNA also exhibited tumour suppressor function in cancer cells. Overexpression of miR-455-3p inhibited tumour growth in prostate cancer^31^ and renal carcinoma cells^32^. In a rat model of thioacetamide-induced hepatocellular carcinoma, decreased expression of miR-455-3p was detected during early stage of cancer development^33^. The same study also identified miR-34a-5p as an early marker for hepatocellular carcinoma although we did not detect any difference in miR-34a-5p between patients with or without LC (Supplementary Table S8). In a multi-omic network analysis, miR-455-3p was identified to be a potential marker for LC^34^ while others had reported the inhibitory effect of miR-455-3p on liver fibrosis^35^.

Low grade inflammation due to chronic HBV infection might progress to liver cirrhosis with hepatocellular carcinoma developing in some carriers^11^. Increased serum miR-122-5p levels had been reported in patients with HBV infection^36,37^ and liver cirrhosis^37^ while decreased liver miR-455-3p expression were detected in advanced liver fibrosis^38^. These discrepant changes in liver tissue and serum suggested that these two miRNAs might be markers for liver damage. However, the exact cellular source and mechanism of their release into circulation require further elucidation. In the miRBase (http://www.mirbase.org/cgi-bin/mirna_entry.pl?acc=MI0016899), the precursor of miR-4532 (stem-loop hsa-mir-4532) was shown to map to the 28 s ribosomal RNA. Taken together, cellular damage during the early stage of LC might cause release of cellular contents including miR-122-5p, miR-455-3p and miR-4532 into the circulation.

In cross-sectional analysis, other researchers had reported increased miR-122-5p and other circulating miRNA^36,39^ in patients with prevalent LC. In our prospective study, we excluded patients with prevalent cancer and detected increased serum levels of miR-122-5p and miR-455–3 in patients during a lead time of zero to four years before the clinical diagnosis of LC. These miRNAs might be sensitive biomarkers of liver damage, we had adjusted for confounders in our multivariate analysis and their risk association with LC remained significant. For other miRNAs identified in prevalent LC cases^17^, we were not able to replicate these findings in our study (Supplementary Table S8) and their validity remained to be verified. The prospective design of our study with adjustment for confounders support the potential utility of miR-122-5p and miR-455-3p as prognostic markers. Whilst experimental studies are needed to characterise their biological functions and targets, this is beyond the scope of the present study.

Circulating miRNA can come from different cell types and tissues, including cell debris from necrotic cells and secreted exosomes and vesicles. Thus, during quantification of serum miRNA levels, it is necessary to develop a strategy to normalize the miRNA level and adjust for background variations in different samples. Unlike cellular mRNA, there is no consensus for serum miRNA normalization. One recommended approach is to compare the serum levels against a fixed quantity of miRNA standard during the qPCR assay^30,40^. However, qPCR assays are microplate-based (96-well and 384-well) assays and performed in batches for hundreds or thousands of samples. This would become very labour intensive if multiple miRNAs are tested with each miRNA requiring a standard curve of its own. Alternatively, we can select a reference miRNA detectable in most samples but with minimal changes in different experimental groups as an internal control for normalization^21^. It has been reported that using only one single gene for normalization could introduce relatively large error in the quantification of gene expression. A better method is to evaluate multiple genes and use the geometric mean of the selected genes for normalization^41,42^. Custom software has been developed to evaluate the expression of the target genes and normalization genes together for the selection of the optimal normalization strategy^43^. Detection of the selected miRNAs in all samples is a prerequisite for the use this multi-gene approach. Given the extracellular nature that serum miRNA comes from the results of different cellular process with different cellular origin, the miRNA selected for use of normalization may not be detectable in all samples. We have tried three miRNAs (miR-186-5p, miR-361-5p and miR-451a) but only miR-186-5p can be detected in most of the samples and used for normalization.

In this study, we adopted a two-step approach to validate the trend of serum miRNA change in T2D-LC patients. We used spike-in controls in multiple steps to establish the increased serum miRNA level of miR-122-5p, miR-455-3p and miR-4532 using qPCR. We applied the same strategy and demonstrated that miR-186-5p was detectable in over 98% of the serum samples with similar levels in patient and control groups. We then used miR-186-5p as the reference gene to normalize for miRNA levels and reported similar trends of increased miRNA levels of miR-122-5p, miR-455-3p and miR-4532 in patients with LC, which were validated in over 2000 patients with T2D using the same reference gene. Of note, reference miRNAs for detecting miRNA associated with colon cancer might show differences in patients with small cell lung cancer and breast cancer^21^. Thus, the use of miR-186-5p as a reference miRNA might be limited to LC only.

This study has several limitations. Firstly, this study used only samples from patients with T2D for the qPCR validation. Increased serum level of miR-122-5p had been reported in diseases with liver damage including HBV infection, cirrhosis^36,40,44^ and LC^36,39^ in general population. Similar information for miR-455-3p was lacking in people without liver disease and/or T2D. Replication of these results in prospective non-T2D cohorts would strengthen the predictive value of these miRNA for detecting early liver cancer in the general population. Secondly, although we established the risk association of LC with increased serum levels of miR-455-3p and miR-122-5p, this was only a relative comparison. We did not establish reference values for serum miR-455-3p and miR-122-5p in patients with or without LC due to the limited number of prevalent and incident LC cases in HKDR. There is a need to incorporate positive miRNA controls to standardize the quantification of miR-455-5p and miR-122-3p using qPCR assays in order to establish reference values for clinical use.

In conclusion, in this well-characterized prospective cohort of patients with T2D, serum miR-455-3p and miR-122-5p independently predicted LC which might be used to select high risk patients with T2D for close surveillance of LC.

## Supplementary Information

Supplementary Information 1.

Supplementary Information 2.

## Data Availability

The data that support the findings of this study are available on request from the corresponding author, Prof. Alice P. S. Kong.
