# Supplementary material for: Detection of increased serum miR-122-5p and miR-455-3p levels before the clinical diagnosis of liver cancer in people with type 2 diabetes
Source: Sci Rep. 2021 Dec 9;11:23756. doi: 10.1038/s41598-021-03222-x (PMC8660865; doi:10.1038/s41598-021-03222-x)
Supplement: Supplementary file 1 — Supplementary Information 1. [file 41598_2021_3222_MOESM1_ESM.docx]

**Supplementary Table S1. Summary of patients with type 2 diabetes (T2D) in stage 2 qPCR validation**

|  | T2D  without cancer | T2D  with  liver cancer | P value |
| --- | --- | --- | --- |
| Cases (N) | 230 | 127 |  |
| Sex (F:M) | 54 : 176 | 27 : 100 |  |
| Age (years) | 59.21 ± 11.50 | 58.59 ± 10.33 | 0.615 |
| Duration of diabetes (years) | 6.12 ± 6.22 | 6.35 ± 5.36 | 0.706 |
| BMI (kg/m^2^) | 24.62 ± 3.49 | 24.21 ± 3.22 | 0.281 |
| HbA1c level (%) | 7.75 ± 1.79 | 7.79 ± 1.79 | 0.840 |
| Fasting plasma glucose (mmol/L) | 8.52 ± 3.28 | 8.42 ± 3.10 | 0.768 |
| Total cholesterol (mmol/L) | 5.20 ± 1.03 | 4.89 ± 1.22 | 0.014 |
| WBC count (10^9^ cells/L) | 7.32 ± 1.87 | 6.65 ± 2.51 | 0.007 |
| Years before cancer diagnosis |  | 6.10 ± 4.89 |  |
| Follow-up period (years) | 16.05 ± 3.53 | 16.12 ± 3.50 | 0.849 |
| All-site cancer risk score | -1.29 ± 0.80 | -0.95 ± 1.05 | 0.002 |
| Tested for HBsAg (%) | 62 (27.0%) | 119 (93.7%) |  |
| Tested positive for HBsAg (%) | 3 (1.3%) | 77 (60.6%) |  |
| Ex- or current use of alcohol (%) | 71 (30.9) | 52 (40.9%) |  |

Data were presented as mean ± standard deviation. The differences between group means were compared by the t-test. Abbreviations: BMI, body mass index; F, female; HbA_1c_, glycated haemoglobin; HBsAg, hepatitis B surface antigen; M, male; N, number; NA, not applicable or not available; T2D, type 2 diabetes; WBC, white blood cell.

**Supplementary Table S2. Summary of patients with type 2 diabetes (T2D) in stage 3 qPCR validation**

|  | T2D  without cancer | T2D  with other cancer | P value |
| --- | --- | --- | --- |
| Cases (N) | 1658 | 487 |  |
| Sex (F:M) | 855 : 803 | 254 : 233 |  |
| Age (years) | 57.48 ± 12.37 | 63.12 ± 10.12 | < 0.001 |
| Duration of diabetes (years) | 7.27 ± 6.75 | 7.17 ± 6.64 | 0.779 |
| BMI (kg/m^2^) | 25.39 ± 4.67 | 25.42 ± 4.22 | 0.905 |
| HbA1c level (%) | 7.63 ± 1.74 | 7.61 ± 1.74 | 0.827 |
| Fasting plasma glucose (mmol/L) | 8.64 ± 3.28 | 8.67 ± 3.29 | 0.875 |
| Total cholesterol (mmol/L) | 5.25 ± 1.17 | 5.19 ± 1.04 | 0.292 |
| WBC count (10^9^ cells/L) | 7.41 ± 3.89 | 7.23 ± 2.04 | 0.356 |
| Years before cancer diagnosis |  | 7.19 ± 5.15 |  |
| Follow-up period (years) | 16.14 ± 3.40 | 16.52 ± 3.36 | 0.028 |
| Cancer risk score | -1.25 ± 0.98 | -1.00 ± 0.92 | < 0.001 |
| Tested for HBsAg (%) | 591 (35.6%) | 198 (40.7%) |  |
| Tested positive for HBsAg (%) | 77 (4.6%) | 15 (3.1%) |  |
| Ex- or current alcohol use (%) | 357 (21.5%) | 110 (22.6%) |  |

Data were presented as mean ± standard deviation. The differences between group means were compared by the t-test. Abbreviations: BMI, body mass index; F, female; HbA_1c_, glycated haemoglobin; HBsAg, hepatitis B surface antigen; M, male; N, number; NA, not applicable or not available; T2D, type 2 diabetes; WBC, white blood cell.

**Supplementary Table S3. List of potential miRNA targets for use as internal control.**

| miRNA | Non-T2D  without cancer | T2D  without cancer | T2D  with liver cancer | Fold Change  (T2D with liver cancer vs T2D without cancer) | P value  (T2D with liver cancer vs. T2D without cancer) | P value  (ANOVA) |
| --- | --- | --- | --- | --- | --- | --- |
| miR-451a | 1.55 ± 0.72 | 1.33 ± 0.70 | 1.32 ± 0.32 | -1.01 | 0.105 | 0.074 |
| miR-191-5p | 1.47 ± 0.49 | 1.26 ± 1.23 | 1.97 ± 1.24 | 1.64 | 0.283 | 0.200 |
| miR-423-5p | 2.51 ± 1.24 | 1.54 ± 0.89 | 1.96 ± 1.33 | 1.33 | 0.218 | 0.243 |
| miR-320a | 6.08 ± 2.06 | 5.71 ± 2.06 | 6.02 ± 1.70 | 1.24 | 0.247 | 0.509 |
| miR-26a-5p | 1.59 ± 0.93 | 1.16 ± 1.08 | 1.34 ± 1.43 | 1.13 | 0.327 | 0.606 |
| miR-361-5p | 1.02 ± 0.32 | 1.01 ± 0.21 | 1.01 ± 0.27 | 1.00 | 0.967 | 0.639 |
| miR-186-5p | 0.89 ± 0.17 | 0.94 ± 0.21 | 0.88 ± 0.18 | -1.04 | 0.967 | 0.986 |

The values were the expression level (mean ± SD) from the microarray study in log2 scale.

**Supplementary Table S4 Summary of microarray results**

| **miRNA** | **Accession** | **Non-DM no cancer*** | **DM no cancer*** | **DM liver cancer*** | **Fold Change (DM liver cancer vs DM no caner)** | **P (DM liver cancer vs no cancer)** | **P (3 groups)** |
| --- | --- | --- | --- | --- | --- | --- | --- |
| hsa-miR-548a-3p | MIMAT0003251 | 2.91 ± 1.10 | 2.35 ± 0.77 | 1.53 ± 0.77 | -1.76 | 0.002 | 3.98E-05 |
| hsa-miR-34c-5p | MIMAT0000686 | 0.83 ± 0.12 | 0.77 ± 0.19 | 1.09 ± 0.33 | 1.25 | 4.30E-05 | 4.46E-05 |
| hsa-mir-5003 | MI0017869 | 0.71 ± 0.16 | 0.85 ± 0.17 | 0.98 ± 0.21 | 1.09 | 0.0065 | 5.05E-05 |
| hsa-miR-383-3p | MIMAT0026485 | 0.94 ± 0.29 | 1.12 ± 0.27 | 0.83 ± 0.17 | -1.22 | 4.41E-05 | 0.0002 |
| hsa-miR-5011-3p | MIMAT0021046 | 0.69 ± 0.12 | 0.82 ± 0.15 | 0.91 ± 0.28 | 1.07 | 0.037 | 0.0002 |
| hsa-miR-7154-5p | MIMAT0028218 | 1.43 ± 0.39 | 1.00 ± 0.25 | 1.26 ± 0.25 | 1.20 | 0.0185 | 0.0003 |
| hsa-miR-122-5p | MIMAT0000421 | 1.12 ± 0.70 | 1.06 ± 1.35 | 2.65 ± 2.87 | 3.01 | 0.0014 | 0.0005 |
| hsa-miR-526b-3p | MIMAT0002836 | 0.80 ± 0.22 | 0.68 ± 0.21 | 0.97 ± 0.17 | 1.23 | 0.0001 | 0.0005 |
| hsa-miR-1287-3p | MIMAT0026738 | 0.79 ± 0.19 | 0.82 ± 0.26 | 1.12 ± 0.23 | 1.23 | 0.0018 | 0.0006 |
| hsa-miR-6081 | MIMAT0023706 | 0.90 ± 0.20 | 0.83 ± 0.23 | 1.12 ± 0.28 | 1.22 | 0.0002 | 0.0006 |
| hsa-miR-192-5p | MIMAT0000222 | 0.74 ± 0.17 | 0.81 ± 0.25 | 1.02 ± 0.30 | 1.16 | 0.0049 | 0.0007 |
| hsa-miR-4779 | MIMAT0019938 | 0.96 ± 0.24 | 1.27 ± 0.29 | 1.03 ± 0.15 | -1.18 | 0.0062 | 0.0009 |
| hsa-miR-4504 | MIMAT0019040 | 0.94 ± 0.13 | 1.10 ± 0.12 | 0.85 ± 0.24 | -1.19 | 0.0007 | 0.0013 |
| hsa-mir-4503 | MI0016866 | 0.96 ± 0.21 | 0.92 ± 0.15 | 1.13 ± 0.24 | 1.16 | 0.001 | 0.0015 |
| hsa-mir-1278 | MI0006425 | 0.97 ± 0.17 | 0.93 ± 0.21 | 1.11 ± 0.15 | 1.13 | 0.0006 | 0.0017 |
| hsa-miR-7106-3p | MIMAT0028110 | 0.91 ± 0.25 | 1.20 ± 0.30 | 0.85 ± 0.27 | -1.27 | 0.0034 | 0.0019 |
| hsa-mir-3156-2 | MI0014230 | 0.84 ± 0.18 | 0.92 ± 0.15 | 1.10 ± 0.26 | 1.13 | 0.0047 | 0.002 |
| hsa-mir-4753 | MI0017392 | 0.89 ± 0.20 | 1.08 ± 0.19 | 0.95 ± 0.2 | -1.09 | 0.016 | 0.0021 |
| hsa-miR-485-5p | MIMAT0002175 | 1.14 ± 0.26 | 1.07 ± 0.23 | 0.90 ± 0.26 | -1.13 | 0.0092 | 0.0025 |
| hsa-mir-1258 | MI0006392 | 1.24 ± 0.21 | 1.23 ± 0.16 | 1.00 ± 0.21 | -1.17 | 0.0033 | 0.0025 |
| hsa-miR-1199-3p | MIMAT0031120 | 1.03 ± 0.24 | 0.69 ± 0.17 | 0.88 ± 0.22 | 1.14 | 0.0053 | 0.0028 |
| hsa-miR-27b-5p | MIMAT0004588 | 0.99 ± 0.22 | 0.84 ± 0.22 | 1.12 ± 0.18 | 1.21 | 0.0008 | 0.0032 |
| hsa-miR-631 | MIMAT0003300 | 1.04 ± 0.21 | 0.91 ± 0.18 | 1.10 ± 0.21 | 1.14 | 0.0045 | 0.0035 |
| hsa-miR-4757-5p | MIMAT0019901 | 0.81 ± 0.19 | 1.04 ± 0.26 | 0.79 ± 0.18 | -1.19 | 0.0023 | 0.004 |
| hsa-miR-1468-3p | MIMAT0026638 | 1.21 ± 0.30 | 1.16 ± 0.35 | 0.97 ± 0.19 | -1.14 | 0.0057 | 0.0043 |
| hsa-miR-7154-3p | MIMAT0028219 | 0.92 ± 0.22 | 0.92 ± 0.23 | 1.11 ± 0.24 | 1.14 | 0.0149 | 0.0048 |
| hsa-miR-95-5p | MIMAT0026473 | 0.80 ± 0.20 | 0.88 ± 0.25 | 1.10 ± 0.26 | 1.16 | 0.0444 | 0.0053 |
| hsa-miR-3179 | MIMAT0015056 | 0.95 ± 0.23 | 1.03 ± 0.29 | 1.22 ± 0.24 | 1.15 | 0.0451 | 0.0053 |
| hsa-miR-4456 | MIMAT0018978 | 0.70 ± 0.18 | 0.62 ± 0.13 | 0.82 ± 0.18 | 1.15 | 0.0016 | 0.0054 |
| hsa-miR-4761-3p | MIMAT0019909 | 0.94 ± 0.18 | 0.97 ± 0.21 | 0.82 ± 0.17 | -1.11 | 0.0014 | 0.0056 |
| hsa-mir-1539 | MI0007260 | 1.21 ± 0.23 | 1.05 ± 0.24 | 1.28 ± 0.21 | 1.18 | 0.0016 | 0.0065 |
| hsa-miR-6735-5p | MIMAT0027371 | 1.93 ± 0.42 | 1.80 ± 0.64 | 1.43 ± 0.38 | -1.29 | 0.0101 | 0.0066 |
| hsa-miR-548f-3p | MIMAT0005895 | 0.96 ± 0.22 | 0.88 ± 0.22 | 1.10 ± 0.24 | 1.16 | 0.0018 | 0.0069 |
| hsa-miR-4307 | MIMAT0016860 | 1.02 ± 0.22 | 0.94 ± 0.21 | 1.06 ± 0.28 | 1.09 | 0.0018 | 0.0069 |
| hsa-miR-409-5p | MIMAT0001638 | 0.97 ± 0.15 | 1.09 ± 0.24 | 0.90 ± 0.16 | -1.14 | 0.002 | 0.0071 |
| hsa-miR-455-3p | MIMAT0004784 | 6.49 ± 1.47 | 6.27 ± 1.84 | 4.58 ± 2.03 | -3.22 | 0.0107 | 0.0075 |
| hsa-mir-4267 | MI0015871 | 1.21 ± 0.27 | 1.19 ± 0.26 | 0.95 ± 0.29 | -1.18 | 0.0119 | 0.0075 |
| hsa-miR-629-5p | MIMAT0004810 | 1.03 ± 0.25 | 0.87 ± 0.20 | 0.98 ± 0.27 | 1.08 | 0.003 | 0.0076 |
| hsa-miR-520f-3p | MIMAT0002830 | 0.89 ± 0.18 | 0.89 ± 0.17 | 1.04 ± 0.24 | 1.11 | 0.0062 | 0.008 |
| hsa-miR-18a-5p | MIMAT0000072 | 0.94 ± 0.20 | 0.95 ± 0.25 | 1.16 ± 0.14 | 1.15 | 0.0061 | 0.0081 |
| hsa-miR-624-3p | MIMAT0004807 | 0.78 ± 0.18 | 0.76 ± 0.18 | 0.93 ± 0.19 | 1.13 | 0.0117 | 0.0082 |
| hsa-miR-4532 | MIMAT0019071 | 6.35 ± 1.32 | 7.23 ± 2.15 | 8.40 ± 1.87 | 2.26 | 0.0117 | 0.0085 |
| hsa-miR-5585-5p | MIMAT0022285 | 0.90 ± 0.22 | 0.88 ± 0.22 | 1.08 ± 0.20 | 1.15 | 0.0066 | 0.0092 |
| hsa-miR-487b-5p | MIMAT0026614 | 0.93 ± 0.17 | 0.74 ± 0.23 | 0.98 ± 0.24 | 1.18 | 0.0028 | 0.0093 |
| hsa-mir-4326 | MI0015866 | 1.02 ± 0.15 | 0.96 ± 0.23 | 1.15 ± 0.27 | 1.14 | 0.0025 | 0.0093 |
| hsa-mir-4767 | MI0017408 | 1.31 ± 0.45 | 0.99 ± 0.32 | 1.38 ± 0.37 | 1.31 | 0.0032 | 0.0096 |
| hsa-miR-302c-3p | MIMAT0000717 | 0.87 ± 0.26 | 0.80 ± 0.20 | 1.05 ± 0.27 | 1.19 | 0.003 | 0.0097 |
| hsa-miR-3672 | MIMAT0018095 | 0.96 ± 0.22 | 1.08 ± 0.24 | 0.94 ± 0.19 | -1.11 | 0.0054 | 0.0097 |
| hsa-mir-4280 | MI0015889 | 0.87 ± 0.20 | 0.89 ± 0.26 | 1.08 ± 0.27 | 1.14 | 0.0476 | 0.0098 |
| hsa-miR-3689a-5p | MIMAT0018117 | 0.91 ± 0.20 | 0.84 ± 0.21 | 1.05 ± 0.20 | 1.15 | 0.0136 | 0.0102 |
| hsa-miR-3689b-5p | MIMAT0018180 | 0.91 ± 0.20 | 0.84 ± 0.21 | 1.05 ± 0.20 | 1.15 | 0.0136 | 0.0102 |
| hsa-miR-3689e | MIMAT0019009 | 0.91 ± 0.20 | 0.84 ± 0.21 | 1.05 ± 0.20 | 1.15 | 0.0136 | 0.0102 |
| hsa-miR-1250-3p | MIMAT0026740 | 0.93 ± 0.22 | 0.92 ± 0.20 | 1.13 ± 0.26 | 1.16 | 0.006 | 0.0103 |
| hsa-miR-518d-3p | MIMAT0002864 | 0.81 ± 0.22 | 0.78 ± 0.16 | 1.00 ± 0.27 | 1.16 | 0.0057 | 0.0106 |
| hsa-miR-1208 | MIMAT0005873 | 0.96 ± 0.25 | 0.75 ± 0.17 | 0.92 ± 0.26 | 1.12 | 0.0496 | 0.0107 |
| hsa-mir-874 | MI0005532 | 0.82 ± 0.17 | 0.92 ± 0.23 | 1.05 ± 0.30 | 1.10 | 0.0204 | 0.012 |
| hsa-mir-5702 | MI0019309 | 0.82 ± 0.18 | 0.85 ± 0.25 | 0.98 ± 0.19 | 1.09 | 0.0214 | 0.0121 |
| hsa-miR-3201 | MIMAT0015086 | 2.55 ± 1.17 | 2.68 ± 1.19 | 1.66 ± 0.98 | -2.02 | 0.005 | 0.0125 |
| hsa-miR-451b | MIMAT0019840 | 0.88 ± 0.16 | 1.06 ± 0.26 | 0.85 ± 0.27 | -1.16 | 0.0174 | 0.0129 |
| hsa-miR-5002-3p | MIMAT0021024 | 1.00 ± 0.28 | 0.92 ± 0.19 | 1.10 ± 0.32 | 1.14 | 0.0038 | 0.013 |
| hsa-miR-939-3p | MIMAT0022939 | 0.92 ± 0.22 | 0.81 ± 0.20 | 1.02 ± 0.38 | 1.16 | 0.0037 | 0.0133 |
| hsa-miR-3655 | MIMAT0018075 | 0.96 ± 0.13 | 1.10 ± 0.34 | 0.85 ± 0.23 | -1.18 | 0.0041 | 0.0136 |
| hsa-mir-642a | MI0003657 | 1.06 ± 0.13 | 1.10 ± 0.34 | 0.95 ± 0.24 | -1.11 | 0.0043 | 0.0142 |
| hsa-miR-2054 | MIMAT0009979 | 0.89 ± 0.19 | 0.80 ± 0.22 | 1.06 ± 0.28 | 1.19 | 0.0042 | 0.0145 |
| hsa-miR-520b | MIMAT0002843 | 0.86 ± 0.26 | 0.88 ± 0.15 | 1.03 ± 0.21 | 1.11 | 0.0105 | 0.0146 |
| hsa-miR-3616-3p | MIMAT0017996 | 1.02 ± 0.28 | 0.87 ± 0.17 | 1.06 ± 0.25 | 1.14 | 0.0058 | 0.0147 |
| hsa-miR-29b-1-5p | MIMAT0004514 | 0.72 ± 0.19 | 0.72 ± 0.21 | 0.89 ± 0.21 | 1.12 | 0.0139 | 0.0149 |
| hsa-miR-513c-3p | MIMAT0022728 | 0.90 ± 0.22 | 0.97 ± 0.23 | 1.11 ± 0.26 | 1.10 | 0.0457 | 0.0149 |
| hsa-miR-24-3p | MIMAT0000080 | 1.04 ± 0.32 | 0.97 ± 0.61 | 1.30 ± 1.16 | 1.26 | 0.0197 | 0.0152 |
| hsa-mir-548o | MI0006402 | 1.01 ± 0.19 | 1.11 ± 0.27 | 0.93 ± 0.15 | -1.13 | 0.0043 | 0.0155 |
| hsa-miR-1180-5p | MIMAT0026735 | 1.83 ± 0.33 | 1.53 ± 0.43 | 1.85 ± 0.32 | 1.25 | 0.0075 | 0.0159 |
| hsa-miR-3129-5p | MIMAT0014992 | 0.77 ± 0.23 | 0.82 ± 0.24 | 1.02 ± 0.26 | 1.15 | 0.0361 | 0.0161 |
| hsa-mir-31 | MI0000089 | 0.86 ± 0.15 | 0.80 ± 0.15 | 0.94 ± 0.29 | 1.10 | 0.0058 | 0.0162 |
| hsa-mir-372 | MI0000780 | 0.87 ± 0.19 | 1.05 ± 0.19 | 0.93 ± 0.18 | -1.09 | 0.0262 | 0.0165 |
| hsa-mir-4526 | MI0016893 | 0.95 ± 0.25 | 1.00 ± 0.17 | 0.80 ± 0.15 | -1.15 | 0.0089 | 0.0169 |
| hsa-mir-4667 | MI0017297 | 0.96 ± 0.19 | 0.89 ± 0.22 | 1.06 ± 0.18 | 1.12 | 0.0048 | 0.0171 |
| hsa-let-7a-2 | MI0000061 | 0.98 ± 0.20 | 1.02 ± 0.23 | 1.15 ± 0.20 | 1.10 | 0.0184 | 0.0174 |
| hsa-mir-302e | MI0006417 | 0.86 ± 0.15 | 0.81 ± 0.24 | 1.06 ± 0.26 | 1.19 | 0.0279 | 0.0175 |
| hsa-mir-5701-1 | MI0019308 | 0.98 ± 0.21 | 0.97 ± 0.21 | 1.14 ± 0.29 | 1.13 | 0.007 | 0.0175 |
| hsa-mir-5701-2 | MI0019593 | 0.98 ± 0.21 | 0.97 ± 0.21 | 1.14 ± 0.29 | 1.13 | 0.007 | 0.0175 |
| hsa-mir-218-2 | MI0000295 | 0.90 ± 0.19 | 1.02 ± 0.22 | 0.86 ± 0.16 | -1.12 | 0.0074 | 0.0181 |
| hsa-miR-6762-3p | MIMAT0027425 | 0.84 ± 0.23 | 0.81 ± 0.28 | 0.98 ± 0.24 | 1.13 | 0.0109 | 0.0184 |
| hsa-miR-655-5p | MIMAT0026626 | 0.78 ± 0.18 | 0.83 ± 0.21 | 1.03 ± 0.24 | 1.15 | 0.025 | 0.0191 |
| hsa-miR-363-3p | MIMAT0000707 | 0.96 ± 0.20 | 0.97 ± 0.23 | 1.07 ± 0.23 | 1.07 | 0.017 | 0.0194 |
| hsa-miR-3616-5p | MIMAT0017995 | 0.89 ± 0.22 | 0.86 ± 0.19 | 1.00 ± 0.25 | 1.10 | 0.0054 | 0.0194 |
| hsa-miR-6744-5p | MIMAT0027389 | 0.87 ± 0.18 | 0.80 ± 0.18 | 1.04 ± 0.32 | 1.18 | 0.0093 | 0.0195 |
| hsa-miR-4679 | MIMAT0019763 | 0.95 ± 0.23 | 0.79 ± 0.20 | 0.92 ± 0.30 | 1.09 | 0.0123 | 0.0203 |
| hsa-miR-5090 | MIMAT0021082 | 1.06 ± 0.24 | 1.05 ± 0.18 | 0.87 ± 0.25 | -1.13 | 0.0303 | 0.0208 |
| hsa-mir-4330 | MI0015902 | 1.02 ± 0.25 | 0.95 ± 0.26 | 1.23 ± 0.21 | 1.21 | 0.0115 | 0.0209 |
| hsa-miR-4283 | MIMAT0016914 | 0.89 ± 0.29 | 0.78 ± 0.14 | 0.91 ± 0.23 | 1.09 | 0.0454 | 0.0222 |
| hsa-miR-657 | MIMAT0003335 | 0.86 ± 0.21 | 0.96 ± 0.31 | 0.73 ± 0.19 | -1.17 | 0.0061 | 0.0224 |
| hsa-mir-34c | MI0000743 | 0.92 ± 0.25 | 0.88 ± 0.20 | 1.09 ± 0.17 | 1.16 | 0.0121 | 0.0226 |
| hsa-miR-4756-5p | MIMAT0019899 | 0.80 ± 0.23 | 0.95 ± 0.27 | 0.76 ± 0.16 | -1.14 | 0.0089 | 0.0237 |
| hsa-miR-938 | MIMAT0004981 | 0.82 ± 0.23 | 0.96 ± 0.24 | 0.73 ± 0.23 | -1.17 | 0.0073 | 0.0241 |
| hsa-miR-17-3p | MIMAT0000071 | 0.81 ± 0.19 | 0.77 ± 0.15 | 0.89 ± 0.21 | 1.09 | 0.0081 | 0.025 |
| hsa-mir-6844 | MI0022690 | 0.94 ± 0.20 | 0.97 ± 0.24 | 1.16 ± 0.20 | 1.14 | 0.0371 | 0.0259 |
| hsa-miR-8054 | MIMAT0030981 | 1.00 ± 0.25 | 0.95 ± 0.22 | 0.89 ± 0.27 | -1.04 | 0.0396 | 0.0268 |
| hsa-miR-5580-3p | MIMAT0022274 | 0.90 ± 0.23 | 0.94 ± 0.23 | 1.14 ± 0.20 | 1.15 | 0.0112 | 0.0269 |
| hsa-mir-4499 | MI0016862 | 0.82 ± 0.18 | 0.85 ± 0.18 | 1.01 ± 0.4 | 1.12 | 0.0345 | 0.0271 |
| hsa-miR-4761-5p | MIMAT0019908 | 0.99 ± 0.28 | 0.92 ± 0.22 | 1.10 ± 0.24 | 1.13 | 0.0092 | 0.0282 |
| hsa-miR-3606-5p | MIMAT0017983 | 0.94 ± 0.21 | 0.89 ± 0.21 | 1.05 ± 0.22 | 1.11 | 0.0109 | 0.0286 |
| hsa-mir-3065 | MI0014228 | 0.88 ± 0.16 | 0.98 ± 0.18 | 0.79 ± 0.22 | -1.14 | 0.0081 | 0.0286 |
| hsa-mir-6861 | MI0022708 | 1.05 ± 0.16 | 1.09 ± 0.14 | 1.16 ± 0.22 | 1.05 | 0.0492 | 0.0292 |
| hsa-mir-451a | MI0001729 | 0.95 ± 0.21 | 0.89 ± 0.27 | 1.16 ± 0.23 | 1.21 | 0.014 | 0.0294 |
| hsa-mir-181b-1 | MI0000270 | 0.94 ± 0.22 | 0.89 ± 0.22 | 1.08 ± 0.23 | 1.13 | 0.0267 | 0.0301 |
| hsa-mir-548ay | MI0022210 | 0.97 ± 0.18 | 0.96 ± 0.20 | 1.07 ± 0.21 | 1.08 | 0.0235 | 0.0303 |
| hsa-mir-4419a | MI0016755 | 0.96 ± 0.30 | 1.02 ± 0.17 | 1.21 ± 0.29 | 1.14 | 0.0186 | 0.0305 |
| hsa-miR-6731-5p | MIMAT0027363 | 0.97 ± 0.22 | 0.85 ± 0.25 | 0.98 ± 0.28 | 1.09 | 0.0097 | 0.0316 |
| hsa-miR-548ao-5p | MIMAT0021029 | 0.93 ± 0.18 | 0.90 ± 0.20 | 1.07 ± 0.22 | 1.13 | 0.0115 | 0.0322 |
| hsa-mir-6889 | MI0022736 | 0.79 ± 0.18 | 0.79 ± 0.19 | 0.93 ± 0.24 | 1.10 | 0.0157 | 0.0327 |
| hsa-miR-548ad | MIMAT0018946 | 0.89 ± 0.16 | 0.84 ± 0.23 | 1.00 ± 0.22 | 1.12 | 0.0102 | 0.033 |
| hsa-mir-633 | MI0003648 | 0.91 ± 0.24 | 0.92 ± 0.16 | 1.04 ± 0.17 | 1.09 | 0.0282 | 0.0333 |
| hsa-mir-621 | MI0003635 | 0.83 ± 0.23 | 0.87 ± 0.19 | 1.03 ± 0.21 | 1.12 | 0.0419 | 0.0336 |
| hsa-miR-3173-3p | MIMAT0015048 | 0.96 ± 0.20 | 0.99 ± 0.27 | 1.13 ± 0.31 | 1.10 | 0.0329 | 0.0343 |
| hsa-miR-592 | MIMAT0003260 | 0.98 ± 0.24 | 0.88 ± 0.22 | 1.06 ± 0.21 | 1.13 | 0.0105 | 0.0353 |
| hsa-mir-96 | MI0000098 | 0.95 ± 0.20 | 0.79 ± 0.18 | 0.98 ± 0.28 | 1.14 | 0.0104 | 0.0354 |
| hsa-miR-6757-3p | MIMAT0027415 | 0.96 ± 0.17 | 1.00 ± 0.19 | 1.16 ± 0.28 | 1.12 | 0.0318 | 0.0363 |
| hsa-miR-3926 | MIMAT0018201 | 1.02 ± 0.23 | 0.97 ± 0.23 | 0.86 ± 0.15 | -1.08 | 0.0489 | 0.0366 |
| hsa-miR-145-5p | MIMAT0000437 | 0.93 ± 0.21 | 0.97 ± 0.26 | 0.82 ± 0.20 | -1.11 | 0.0238 | 0.0368 |
| hsa-miR-338-3p | MIMAT0000763 | 0.78 ± 0.22 | 0.75 ± 0.17 | 0.88 ± 0.32 | 1.10 | 0.0152 | 0.0369 |
| hsa-miR-3617-5p | MIMAT0017997 | 0.97 ± 0.18 | 0.93 ± 0.20 | 1.05 ± 0.3 | 1.08 | 0.0115 | 0.0376 |
| hsa-mir-27a | MI0000085 | 0.93 ± 0.19 | 0.90 ± 0.18 | 0.99 ± 0.21 | 1.06 | 0.0471 | 0.038 |
| hsa-mir-642a | MI0003657 | 1.00 ± 0.29 | 1.05 ± 0.25 | 0.84 ± 0.21 | -1.15 | 0.0156 | 0.0388 |
| hsa-miR-1539 | MIMAT0007401 | 1.01 ± 0.28 | 0.91 ± 0.25 | 0.98 ± 0.34 | 1.04 | 0.0122 | 0.0398 |
| hsa-miR-3127-3p | MIMAT0019201 | 1.04 ± 0.19 | 1.06 ± 0.18 | 0.93 ± 0.21 | -1.10 | 0.0354 | 0.0399 |
| hsa-miR-4724-5p | MIMAT0019841 | 1.03 ± 0.27 | 1.03 ± 0.21 | 1.13 ± 0.32 | 1.07 | 0.0489 | 0.0405 |
| hsa-miR-570-5p | MIMAT0022707 | 1.24 ± 0.38 | 1.06 ± 0.29 | 1.29 ± 0.31 | 1.17 | 0.015 | 0.0407 |
| hsa-miR-548ai | MIMAT0018989 | 1.24 ± 0.38 | 1.06 ± 0.29 | 1.29 ± 0.31 | 1.17 | 0.015 | 0.0407 |
| hsa-miR-511-5p | MIMAT0002808 | 0.77 ± 0.20 | 0.74 ± 0.17 | 0.94 ± 0.27 | 1.14 | 0.0143 | 0.0415 |
| hsa-miR-8070 | MIMAT0030997 | 1.05 ± 0.19 | 0.84 ± 0.20 | 0.99 ± 0.22 | 1.11 | 0.0452 | 0.0419 |
| hsa-miR-219a-5p | MIMAT0000276 | 0.90 ± 0.20 | 1.01 ± 0.30 | 0.84 ± 0.25 | -1.12 | 0.0337 | 0.042 |
| hsa-mir-4632 | MI0017259 | 0.97 ± 0.19 | 1.01 ± 0.20 | 1.20 ± 0.35 | 1.14 | 0.0417 | 0.042 |
| hsa-mir-4439 | MI0016782 | 0.98 ± 0.29 | 1.09 ± 0.20 | 0.91 ± 0.20 | -1.13 | 0.0125 | 0.0427 |
| hsa-mir-4775 | MI0017418 | 0.97 ± 0.34 | 1.10 ± 0.32 | 0.95 ± 0.19 | -1.11 | 0.0128 | 0.044 |
| hsa-miR-490-5p | MIMAT0004764 | 0.72 ± 0.17 | 0.74 ± 0.17 | 0.79 ± 0.41 | 1.04 | 0.0358 | 0.0444 |
| hsa-miR-633 | MIMAT0003303 | 0.86 ± 0.24 | 1.10 ± 0.25 | 0.89 ± 0.24 | -1.16 | 0.0488 | 0.0457 |
| hsa-mir-7705 | MI0025241 | 1.04 ± 0.24 | 0.94 ± 0.24 | 1.15 ± 0.18 | 1.15 | 0.0149 | 0.0462 |
| hsa-mir-3199-2 | MI0014248 | 1.00 ± 0.18 | 0.94 ± 0.17 | 1.03 ± 0.23 | 1.07 | 0.0138 | 0.047 |
| hsa-miR-2115-5p | MIMAT0011158 | 0.85 ± 0.23 | 0.76 ± 0.18 | 0.92 ± 0.26 | 1.12 | 0.022 | 0.0474 |
| hsa-miR-8058 | MIMAT0030985 | 0.81 ± 0.22 | 0.81 ± 0.23 | 0.98 ± 0.16 | 1.13 | 0.0186 | 0.0482 |
| hsa-mir-6510 | MI0022222 | 0.93 ± 0.27 | 0.92 ± 0.17 | 1.04 ± 0.20 | 1.09 | 0.0255 | 0.0488 |
| hsa-miR-4782-5p | MIMAT0019944 | 0.81 ± 0.18 | 0.74 ± 0.31 | 0.95 ± 0.17 | 1.16 | 0.0273 | 0.0489 |
| hsa-mir-6824 | MI0022669 | 1.00 ± 0.23 | 1.06 ± 0.19 | 0.88 ± 0.21 | -1.13 | 0.0174 | 0.0493 |
| hsa-miR-4641 | MIMAT0019701 | 0.98 ± 0.26 | 1.09 ± 0.26 | 0.91 ± 0.24 | -1.14 | 0.0206 | 0.0494 |
| hsa-mir-595 | MI0003607 | 0.87 ± 0.20 | 0.82 ± 0.12 | 0.95 ± 0.27 | 1.10 | 0.018 | 0.0499 |

* The values are mean ± standard deviation in log2 scale.

**Supplementary Table S5. Summary of stage 2 qPCR validation results**

| **miRNA** | **T2D**  **without cancer** | **T2D**  **with liver cancer** | **Fold change** | **P value**  **(t-test)** |
| --- | --- | --- | --- | --- |
| *Adjusted by spike-in control and positive RNA control* | | | | |
| miR-122-5p | 25948.0 ± 8532. | 254229.5 ± 5893.5 | 9.80 | < 0.001* |
| miR-455-3p | 146.0 ± 53.8 | 3547.1 ± 1121.3 | 24.30 | 0.003* |
| miR-4532 | 13152.1 ± 1606.6 | 45288.5 ± 19784.6 | 3.44 | 0.108* |
| miR-548a-3p | 836.9 ± 148.5 | 965.3 ± 219.7 | 1.15 | 0.619 |
| miR-3201 | 1131.2 ± 500.7 | 1711.2 ± 551.2 | 1.51 | 0.463 |
| miR-186-5p | 18990.0 ± 2908.3 | 16529.7 ± 3817.5 | 0.87 | 0.612 |
|  | | | | |
| *Normalized by the level of miR-186-5p* | | | | |
| miR-122-5p | 7.106 ± 2.408 | 99.892 ± 30.337 | 14.06 | 0.003* |
| Log of miR-122-5p | -0.229 ± 0.063 | 1.185 ± 0.073 |  | < 0.001 |
| miR-455-3p | 4.945 ± 2.420 | 9.385 ± 5.853 | 1.90 | 0.415 |
| Log of miR-455-3p | -2.636 ± 0.102 | -1.425 ± 0.138 |  | < 0.001 |
| miR-4532 | 525.403 ± 195.143 | 3805.619 ± 2888.060 | 7.24 | 0.255* |
| Log of miR-4532 | 0.217 ± 0.085 | 0.677 ± 0.114 |  | 0.001 |
| miR-548a-3p | 50.937 ± 24.187 | 48.444 ± 25.549 | 0.95 | 0.948 |
| Log of miR-548a-3p | -1.210 ± 0.082 | -1.060 ± 0.125 |  | 0.303 |
| miR-3201 | 75.364 ± 32.066 | 110.807 ± 44.591 | 1.47 | 0.516 |
| Log of miR-3201 | -1.178 ± 0.093 | -0.833 ± 0.132 |  | 0.031 |

Data were presented as mean ± standard deviation. * Unpaired t-test with Welch’s correction.

**Supplementary Table S6 Summary of serum miRNA level and HBsAg status**

| **miRNA** | **HBsAg**  **Positive** | **HBsAg**  **negative** | **Not tested for HBsAg** | **P value**  **(HBsAg +ve vs. HBsAg -ve)** |
| --- | --- | --- | --- | --- |
| **miR-122-5p** |  |  |  |  |
| T2D without cancer | 80.808 ± 23.720  (N = 76) | 23.008 ± 4.577  (N = 514) | 34.078 ± 4.235  (N = 1166) | 0.019* |
| T2D liver cancer | 108.890 ±34.670  (N = 75) | 24.588 ± 5.223  (N = 37) | 125.371 ± 114.138  (N = 8) | 0.019* |
| T2D other cancer | 122.091 ± 103.096  (N = 15) | 52.923 ± 30.431  (N = 176) | 24.327 ± 6.374  (N = 269) | 0.525 |
| Total | 97.226 ± 21.062 | 30.330 ± 8.050 | 32.767 ± 3.676 | 0.003* |
|  |  |  |  |  |
| **miR-455-3p** |  |  |  |  |
| T2D without cancer | 1.536 ± 1.222  (N = 74) | 0.903 ± 0.436  (N = 497) | 1.038 ± 0.423  (N = 1049) | 0.606 |
| T2D liver cancer | 2.384 ± 1.465  (N = 77) | 25.252 ± 18.604  (N = 31) | 1.391 ± 0.735  (N = 7) | 0.229 |
| T2D other cancer | 0.226 ± 0.121  (N = 13) | 1.011 ± 0.662  (N =152) | 0.331 ± 0.091  (N = 232) | 0.730 |
| Total | 1.802 ± 0.867 | 2.139 ± 0.999 | 0.912 ± 0.345 | 0.874 |
|  |  |  |  |  |
| **miR-4532** |  |  |  |  |
| T2D without cancer | 1066.620 ± 1051.158  (N = 80) | 445.574 ± 279.603  (N = 568) | 190.850 ± 45.745  (N =1226) | 0.461 |
| T2D liver cancer | 5374.480 ± 4648.887  (N = 84) | 1609.363 ± 1001.787  (N = 31) | 30.570 ± 16.103  (N = 8) | 0.566 |
| T2D other cancer | 14.175 ± 7.374  (N = 15) | 146.379 ± 64.936  (N =181) | 211.937 ± 115.482  (N = 289) | 0.559 |
| Total | 2888.905 ± 2123.076 | 434.449 ± 208.158 | 194.009 ± 42.835 | 0.251 |

Data were presented as mean ± standard deviation. * Unpaired t-test with Welch’s correction.

**Supplementary Table S7 Summary of serum miRNA level and alcohol use in patients with type 2 diabetes**

| **miRNA** | **Alcohol user** | **Non-alcohol user** | **P value** |
| --- | --- | --- | --- |
| **miR-122-5p** |  |  |  |
| T2D without cancer | 32.276 ± 8.021  (N =393) | 33.187 ± 3.572  (N = 1355) | 0.908 |
| T2D liver cancer | 83.144 ± 42.615  (N = 50) | 84.603 ± 25.654  (N = 70) | 0.975 |
| T2D other cancer | 26.651 ± 9.851  (N =103) | 42.149 ± 16.256  (N = 353) | 0.613 |
| Total | 35.874 ± 7.218 | 36.991 ± 4.343 | 0.899 |
|  |  |  |  |
| **miR-455-3p** |  |  |  |
| T2D without cancer | 0.936 ± 0.562  (N = 372) | 1.051 ± 0.368  (N = 1239) | 0.877 |
| T2D liver cancer | 7.359 ± 3.495  (N = 48) | 10.960 ± 10.062  (N = 62) | 0.762 |
| T2D other cancer | 0.160 ± 0.046  (N = 92) | 0.724 ± 0.340  (N = 302) | 0.361 |
| Total | 1.399 ± 0.530 | 1.373 ± 0.486 | 0.977 |
|  |  |  |  |
| **miR-4532** |  |  |  |
| T2D without cancer | 536.759 ± 372.456  (N = 426) | 238.855 ± 70.316  (N = 1439) | 0.432* |
| T2D liver cancer | 7846.072 ± 7034.196  (N = 50) | 1084.474 ± 701.048  (N = 73) | 0.343* |
| T2D other cancer | 50.193 ± 18.907  (N = 110) | 222.154 ± 95.096  (N = 371) | 0.326 |
| Total | 1069.086 ± 659.003 | 268.347 ± 63.092 | 0.227* |

Data were presented as mean ± standard deviation. * Unpaired t-test with Welch’s correction.

**Supplementary Table S8 Serum levels of previous reported liver cancer related miRNA from the stage 1 microarray study**

| **miRNA** | **No T2D**  **No cancer** | **T2D**  **No cancer** | **T2D**  **Liver cancer** | **Fold Change**  **(T2D Liver cancer vs T2D No cancer)** | **P value**  **(T2D Liver cancer vs T2D No cancer)** | **ANOVA**  **P** |
| --- | --- | --- | --- | --- | --- | --- |
| **miR-122-5p** | 1.12 ± 0.70 | 1.06 ± 1.35 | 2.65 ± 2.87 | 3.01 | **0.0014** | **0.0005** |
| **miR-192a-5p** | 0.74 ± 0.17 | 0.81 ± 0.25 | 1.02 ± 0.30 | 1.16 | **0.0049** | **0.0007** |
| **miR-223-3p** | 0.91 ± 0.28 | 1.04 ± 0.29 | 1.03 ± 0.26 | -1.00 | 0.9896 | 0.4941 |
| **miR-21-5p** | 0.84 ± 0.23 | 0.87 ± 0.27 | 0.86 ± 0.21 | -1.01 | 0.5660 | 0.5670 |
| **miR-27a-3p** | 0.93 ± 0.24 | 0.99 ± 0.30 | 0.89 ± 0.34 | -1.07 | 0.5289 | 0.7826 |
| **miR-26a-5p** | 1.59 ± 0.93 | 1.16 ± 1.08 | 1.34 ± 1.43 | 1.13 | 0.3266 | 0.6058 |
| **miR-34a-5p** | 0.85 ± 0.13 | 0.81 ± 0.22 | 0.80 ± 0.31 | -1.01 | 0.8647 | 0.9447 |

The values were the expression level (mean ± SD) from the microarray study in log2 scale.
