# Supplementary material for: Detection of increased serum miR-122-5p and miR-455-3p levels before the clinical diagnosis of liver cancer in people with type 2 diabetes
Source: Sci Rep. 2021 Dec 9;11:23756. doi: 10.1038/s41598-021-03222-x (PMC8660865; doi:10.1038/s41598-021-03222-x)
Supplement: Supplementary file 2 — Supplementary Information 2. [file 41598_2021_3222_MOESM2_ESM.docx]

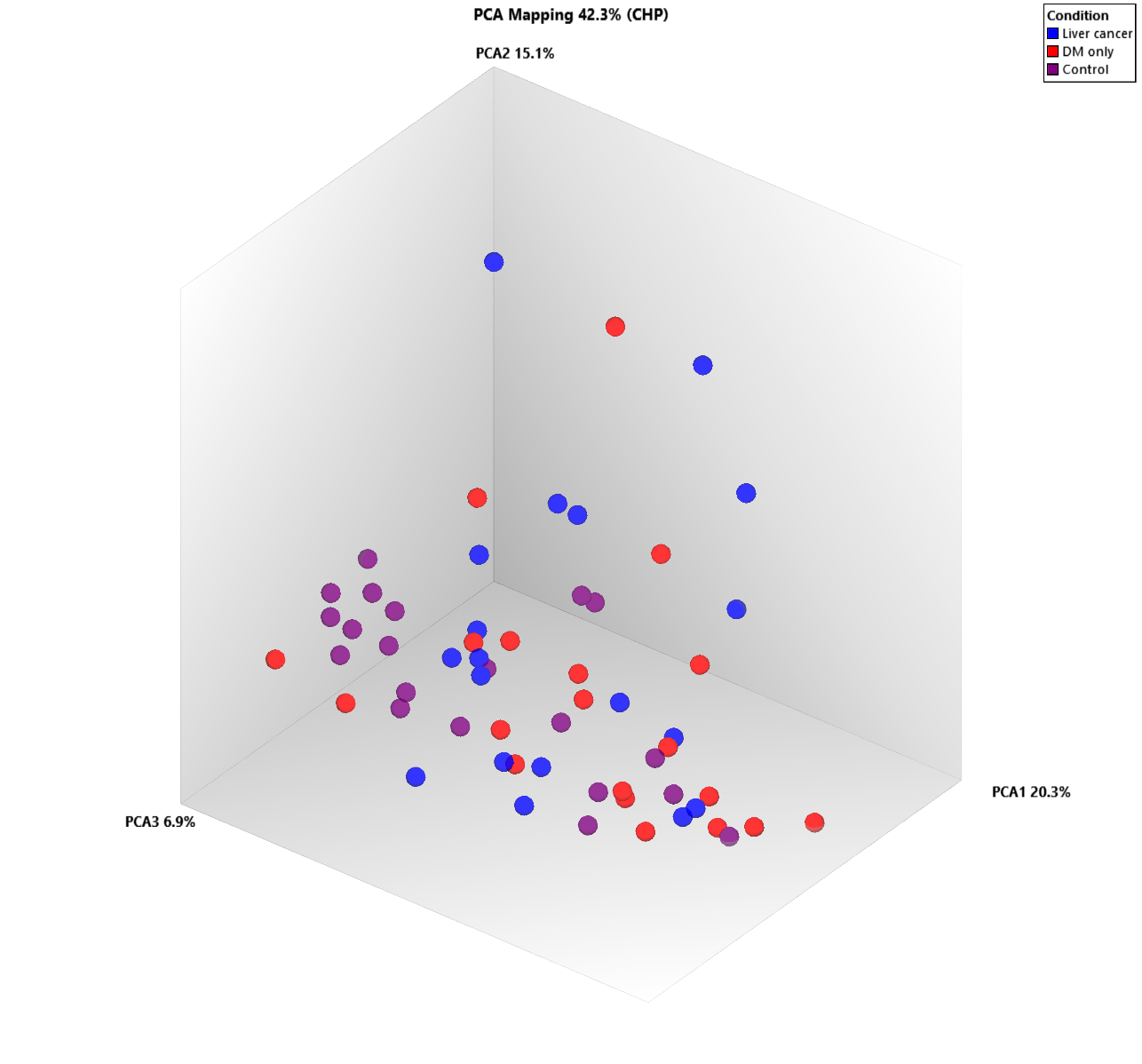


**Supplementary Figure S1**. Principle component analysis (PCA) of the microarray result.


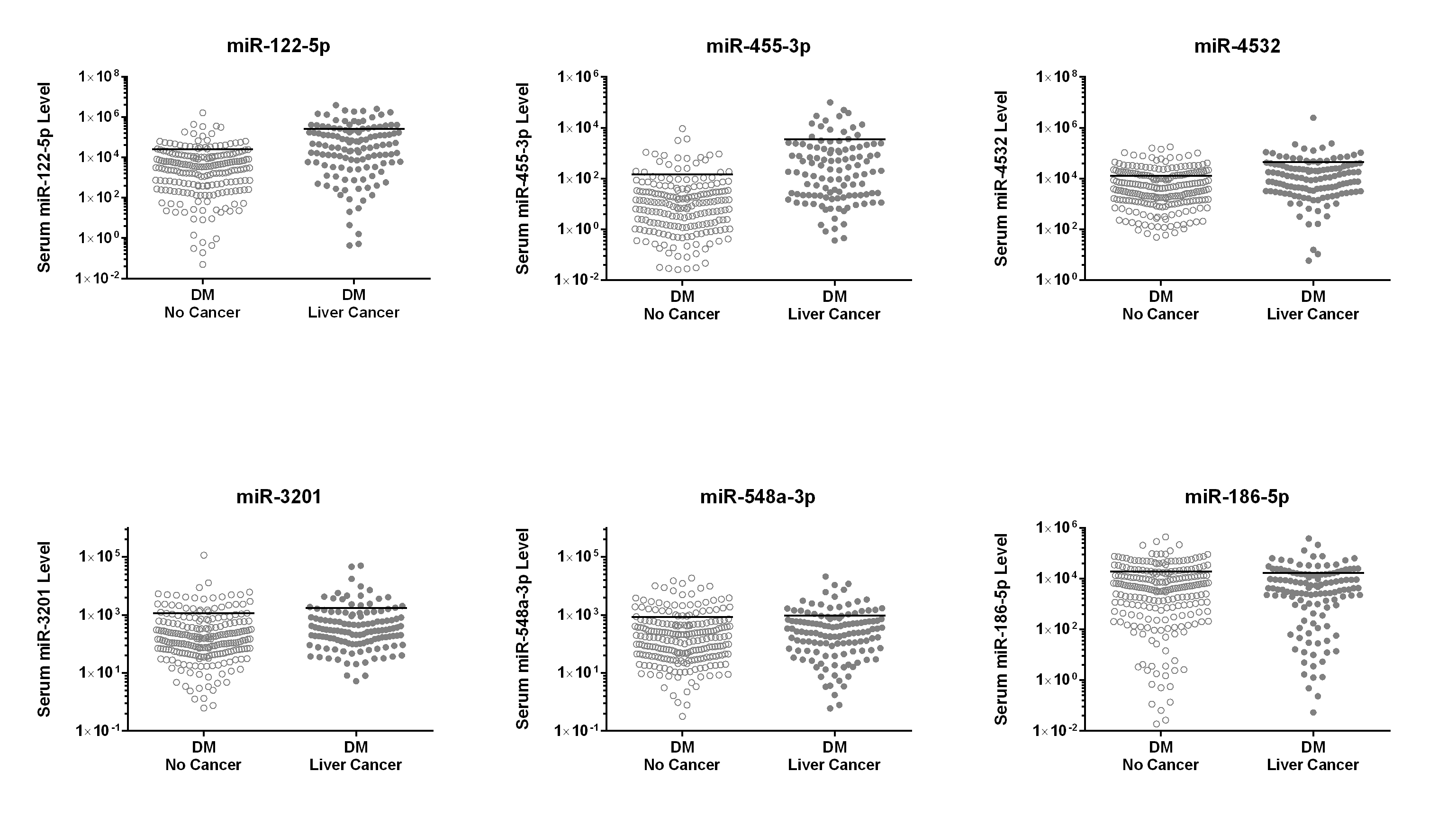


**Supplementary Figure S2**. Quantification of selected miRNA in the serum of DM no cancer and DM liver cancer patients. The miRNA tested was shown on the top of each panel. The horizontal line represented the mean of the group.


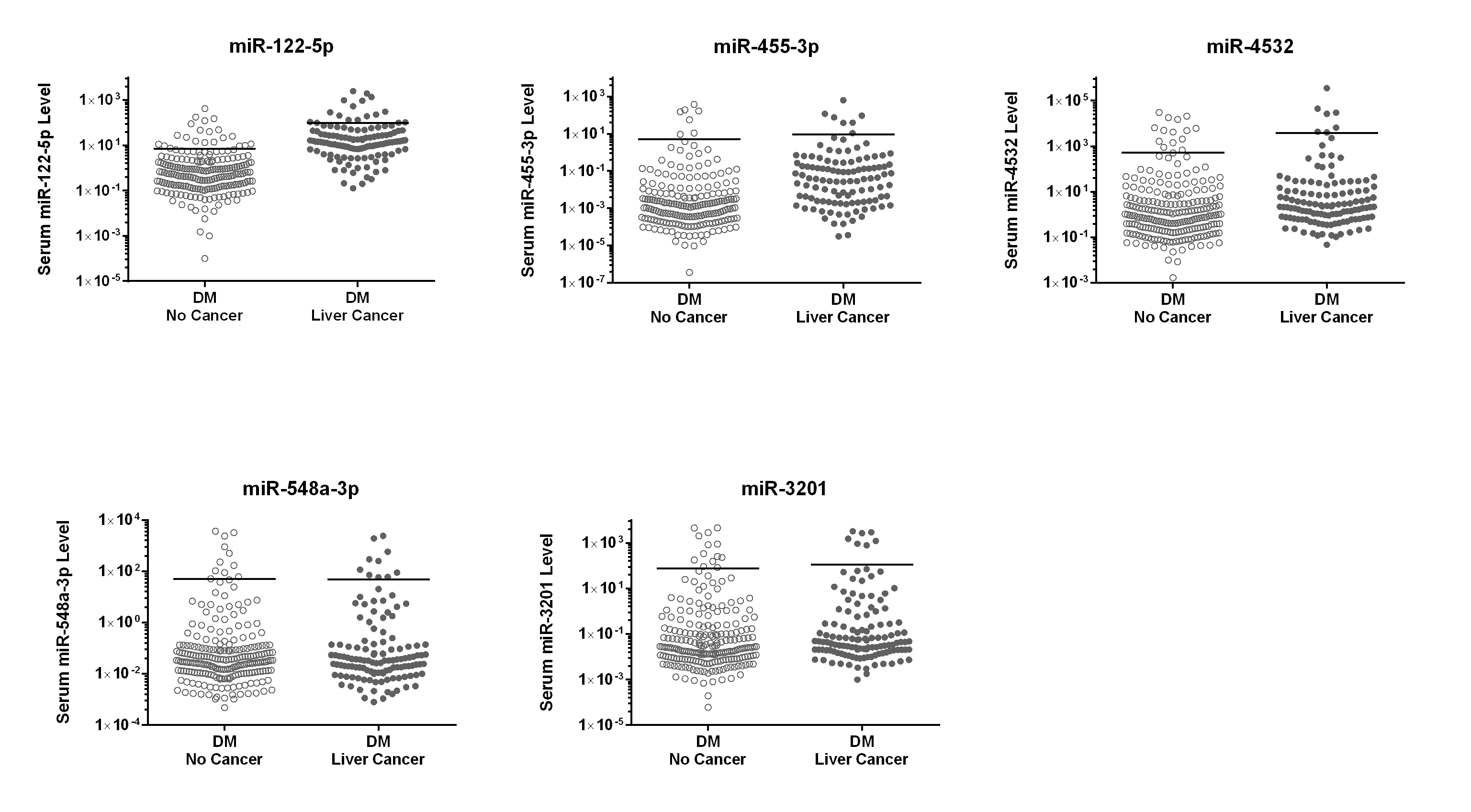


**Supplementary Figure S3**. Quantification of selected miRNA in the serum of DM no cancer and DM liver cancer patients using serum miR-186-5p as internal control. The miRNA tested was shown on the top of each panel. The serum miRNA levels were normalized to the serum levels of miR-186-5p. The horizontal line represented the mean of the group.
